# Increased ADORA1 expression in pancreatic islets coincides with insulin secretory defects in human type 2 diabetes

**DOI:** 10.1016/j.jbc.2026.113501

**Published:** 2026-08-27

**Authors:** Israa Mohammed Al-Amily, Fariborz Parandeh, Stefan Amisten, Pontus Dunér, Sandra Meidute-Abaraviciene, Eva Grapengiesser, Bo Hellman, Albert Salehi

**Affiliations:** 1 Department of Clinical Science, Malmö, Division of Islet Cell Physiology, University of Lund, Lund, Sweden; 2 Experimental Cardiovascular Research, University of Lund, Lund, Sweden; 3 Department of Medical Cell Biology, Uppsala University, Uppsala, Sweden

**Keywords:** G-protein coupled receptors, GPCRs, cAMP/PKA system, gene expression, metabolic diseases

## Abstract

Adenosine receptors (ADORAs) are G-protein coupled receptors that critically modulate cell function, yet their specific roles in regulating insulin and glucagon secretion remain incompletely understood. While RNA sequencing of isolated human islets, confirmed by qPCR, revealed that only ADORA1 and ADORA2A transcripts are abundantly expressed, mouse islets express all four receptor subtypes (Adora1, Adora2a, Adora2b, and Adora3). Confocal immunofluorescence demonstrated ADORA1 protein localization in both islet β- and α-cells. Transcriptome correlation analysis of human islets identified 87 genes strongly associated with ADORA1 expression, enriched in pathways regulating carbohydrate and lipid metabolism, cell cycle, apoptosis, proliferation, endocrine system development, and metabolic disease. ADORA1 mRNA levels were positively correlated with HbA1c and elevated in islets of type 2 diabetes (T2D) donors, implicating ADORA1 in β-cell dysfunction. In *ob/ob* mouse islets, reduced Adora1 expression coincided with enhanced pulsatile insulin secretion, while the control islets showed a biphasic with a weaker second phase insulin secretory response. Adenosine efficiently suppressed insulin secretion in both *ob/ob* and control islets. ADORA1 antagonist DPCPX attenuated adenosine-induced suppression of insulin release. Adora1-KD mice exhibited a restored, pulsatile second-phase insulin response absent in controls. In human islets, adenosine suppressed both glucose-stimulated insulin secretion and cAMP generation, effects prevented by DPCPX. Adenosine also impaired β-cell viability and proliferation, which were rescued by ADORA1 inhibition. Collectively, our findings identify ADORA1 as a key inhibitory modulator of β-cell function under diabetogenic stress, thereby a promising therapeutic target to preserve insulin secretory capacity in early T2D.

It is well documented that insulin secretion is a highly regulated multistep biological process whereby pancreatic β-cells respond to metabolic, neuronal, or hormonal stimuli to produce and secrete insulin (1). The precise control of glucose-stimulated insulin secretion (GSIS) represents a critical component of glucose homeostasis, with disruption of this process contributing to the pathogenesis of type 2 diabetes (T2D) (2, 3, 4, 5).

Although glucose is the primary physiological secretagogue for insulin secretion, its action is finely modulated by numerous autocrine, paracrine, neuronal, and hormonal signals acting through G protein-coupled receptors (GPCRs) expressed on pancreatic β-cells (6, 7, 8, 9). These GPCRs regulate intracellular signaling pathways that either potentiate or suppress GSIS, thereby contributing to the maintenance of glucose homeostasis (6, 7). Notably, activation of Gi/o-coupled GPCRs suppresses adenylyl cyclase activity, leading to reduced intracellular cyclic AMP (cAMP) levels and impaired β-cell function (6, 8). Persistent inhibition of β-cell secretory function by Gi/o-coupled GPCRs would ultimately contribute to glucose intolerance, chronic hyperglycemia, and the development of T2D-like metabolic disturbances (10). Among the endogenous inhibitory modulators of β-cell function, adenosine has emerged as an important suppressor of insulin release (11, 12).

Adenosine functions as an autacoid, an endogenous metabolite that plays a critical role in regulating vital body functions, including pancreatic islet hormone secretion (11, 12). Unlike its intracellular concentration in the millimolar (mM) range, where adenosine serves as a substrate in cellular metabolism, adenosine is released into the extracellular space or generated through metabolism of extracellular adenosine triphosphate (ATP) within pancreatic islets at nanomolar to micromolar (nM-μM) concentrations, where it functions as a signaling molecule (13). In this extracellular environment, adenosine acts as an autocrine and paracrine signaling molecule, exerting regulatory effects on pancreatic insulin and glucagon secretion (11, 12, 14). We have previously demonstrated that adenosine exerts an inhibitory effect on insulin secretion, although the complete underlying mechanism(s) remained incompletely defined (11, 12).

Adenosine mediates its widespread physiological and pathophysiological actions through four distinct seven transmembrane-spanning G-protein-coupled adenosine receptor subtypes, classified as Adora1 (A1R), Adora2A (A2AR), Adora2B (A2BR), and Adora3 (A3R) (15). Each receptor subtype couples to distinct intracellular signaling cascades in a receptor-specific and cell-type-dependent manner (15). While ADORA1 and ADORA3 are coupled to inhibitory G_i/o_ proteins that suppress adenylyl cyclase activity and reduce intracellular cyclic adenosine monophosphate (cAMP) levels, ADOR2A and ADORA2B are coupled to stimulatory G_s_ proteins that activate adenylyl cyclase and increase cAMP production (15, 16). Since ADORA3 is absent in human β-cells (6, 7), and since ADORA1 is the predominant inhibitory adenosine receptor expressed in human pancreatic islets and provides the most direct mechanistic link between extracellular adenosine, suppression of cAMP signaling, and impaired β-cell function (11), in the present study, we focused on the ADORA1 expression and its relation to hyperglycemic and diabetic conditions.

ADORA1 is widely distributed throughout the central and peripheral nervous systems, as well as on several non-neuronal cell types, including pancreatic β-cells (11, 17). ADORA1 agonists and antagonists exert neuromodulatory effects in various centers, leading to sedation, analgesia, vasodilation, and bradycardia with minimal effects on the respiratory drive, thereby accounting for their favorable safety profile (15, 18). Beyond its central nervous system actions, adenosine signaling represents an important regulator of metabolic processes, including glucose homeostasis, insulin secretion, and glucagon release (12, 18).

We have previously shown that adenosine negatively modulates insulin secretion, and Adora1 knockdown in mice is associated with potentiation of GSIS (11). Notably, conflicting data exist in the literature, with some studies reporting minimal or negligible effects of adenosine on insulin secretion (9, 11, 16), suggesting that the role of adenosine in regulating β-cell function may be context-dependent or influenced by experimental conditions.

While insulin secretion has been extensively characterized and numerous regulatory pathways involving nutrients, incretins, and neurotransmitters have been established (19, 20, 21, 22), glucagon secretion remains less well understood. Glucagon release is complex and subject to differential regulation by glucose levels (23, 24) and inhibition by insulin released from β-cells (25, 26, 27). Furthermore, recent evidence demonstrates that intra-islet adenosine plays an unexpected role in promoting glucagon release *via* activation of α-cell Gs-coupled A2A adenosine receptors, suggesting a sophisticated interplay between adenosine signaling and islet hormone secretion (28). Accordingly, we found it valuable to examine the effects of adenosine on glucagon release in parallel with insulin secretion in the present study.

The negative impact of adenosine signaling on the β-cell function suggests that selective targeting of adenosine receptors using subtype-specific agonists and antagonists could have important therapeutic implications for metabolic disorders and diabetes (11, 15, 18). This therapeutic potential is particularly relevant given the mounting evidence that dysregulation of adenosine signaling contributes to impaired glucose tolerance and insulin resistance in type 2 diabetes (18).

The present study aimed to examine the effects of adenosine on intracellular Ca^2+^ dynamics and pulsatility of insulin secretion in the diabetic *ob/ob* mouse model, as well as to investigate insulin secretion in relation to ADORA1 activation in isolated human pancreatic islets obtained from both non-diabetic and type 2 diabetic donors. Additionally, we employed Adora1 knockdown (Adora1-KD) mice to dissect the specific contribution of Adora1 signaling to glucose-stimulated hormone secretion and to evaluate whether Adora1 represents a viable therapeutic target for modulating islet function.

## Results

### Expression of ADORA1 in pancreatic islets

First, we re-examined our previously published RNA sequencing (RNA-seq) data from isolated human pancreatic islets (29, 30) to compare ADORA1 expression with that of other adenosine receptors. The RNA-seq dataset is available at https://www.pnas.org/doi/10.1073/pnas.1402665111 and GEO (GSE50398) (30). The analysis showed that ADORA1 and ADORA2A transcripts are among the most abundant adenosine receptor transcripts in islets, with expression levels significantly higher than those of ADORA2B and ADORA3 (Fig. 1*A*).

**Figure 1:**
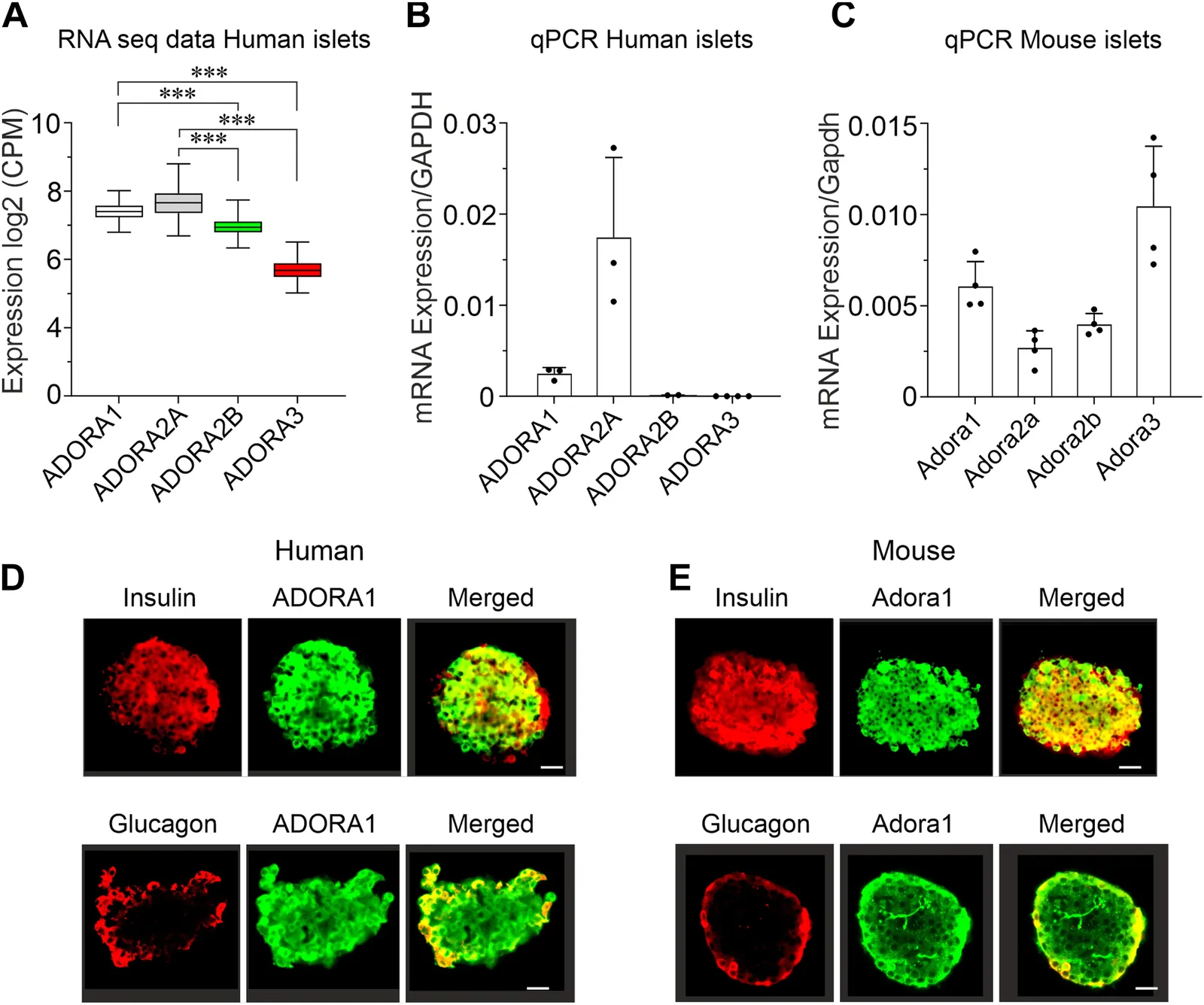
**Adenosine receptors in human and mouse islets.***A*, ADORA1, ADORA2A, AdORA2B, and ADORA3 transcript expression profile in isolated human pancreatic islets (n = 89) analyzed by RNA seq. Box plots represent ADORA1, ADORA2A, AdORA2B, and ADORA3 transcript expression. ∗∗∗*p* < 0.001 (unpaired Student *t* test). *B*, analysis of ADORA1, ADORA2A, AdORA2B and ADORA3 mRNA expression in isolated human pancreatic islets by qPCR (n = 4). *C*, analysis of Adora1, Adora2A, Adora2B and Adora3 mRNA expression in isolated mouse pancreatic islets by qPCR (n = 4). *D*, confocal microscopy images of ADORA1 expression in human (n = 4) and in mouse (n = 4) pancreatic islets (*E*) co-stained with insulin or glucagon. Bar indicates 10 μm.

Because automated, genome-wide approaches such as RNA-seq can occasionally yield false-positive or false-negative detection of individual genes among the ∼15,017 analyzed genes, quantitative PCR (qPCR) was used to validate the RNA-seq-derived expression of ADORA1, ADORA2A, ADORA2B, and ADORA3 mRNAs in human and mouse islets. Consistent with the RNA-seq data, qPCR detected robust expression of ADORA1 and ADORA2A, whereas ADORA2B and ADORA3 mRNA levels were very low or at the limit of detection in human islets (Fig. 1*B*). Figure 1*C* shows the expression profiles of Adora1, Adora2a, Adora2b, and Adora3 in isolated mouse islets.

Immunohistochemistry was then used to examine ADORA1 protein expression in insulin-producing β-cells and glucagon-producing α-cells in human islets. Double immunolabeling combined with confocal microscopy demonstrated prominent ADORA1 staining in both insulin-positive β-cells and glucagon-positive α-cells in human pancreatic islets (Fig. 1*D*). A similar pattern of Adora1 protein expression was observed in insulin-positive β-cells and glucagon-positive α-cells in mouse pancreatic islets (Fig. 1*D*).

RNA-seq analysis of human islets revealed that a large number of gene transcripts correlate with ADORA1 expression. Using a stringent correlation cut-off (r^2^ > 0.70), the mRNA levels of 88 genes were found to be strongly associated with ADORA1 mRNA expression in human islets (Table S1). Ingenuity Pathway Analysis (IPA, Qiagen) was used to functionally annotate these 87 genes, with a focus on identifying genes linked to β-cell function/mass, proliferation, and apoptosis. IPA identified 70 functional annotations, of which 53 genes were assigned to seven biological functions of direct relevance to β-cell mass and glucose homeostasis, including carbohydrate and lipid metabolism, cell cycle, cell death and survival, cellular growth and proliferation, endocrine system development and function, endocrine system disorders, and metabolic disease (Fig. 2*A*).

**Figure 2:**
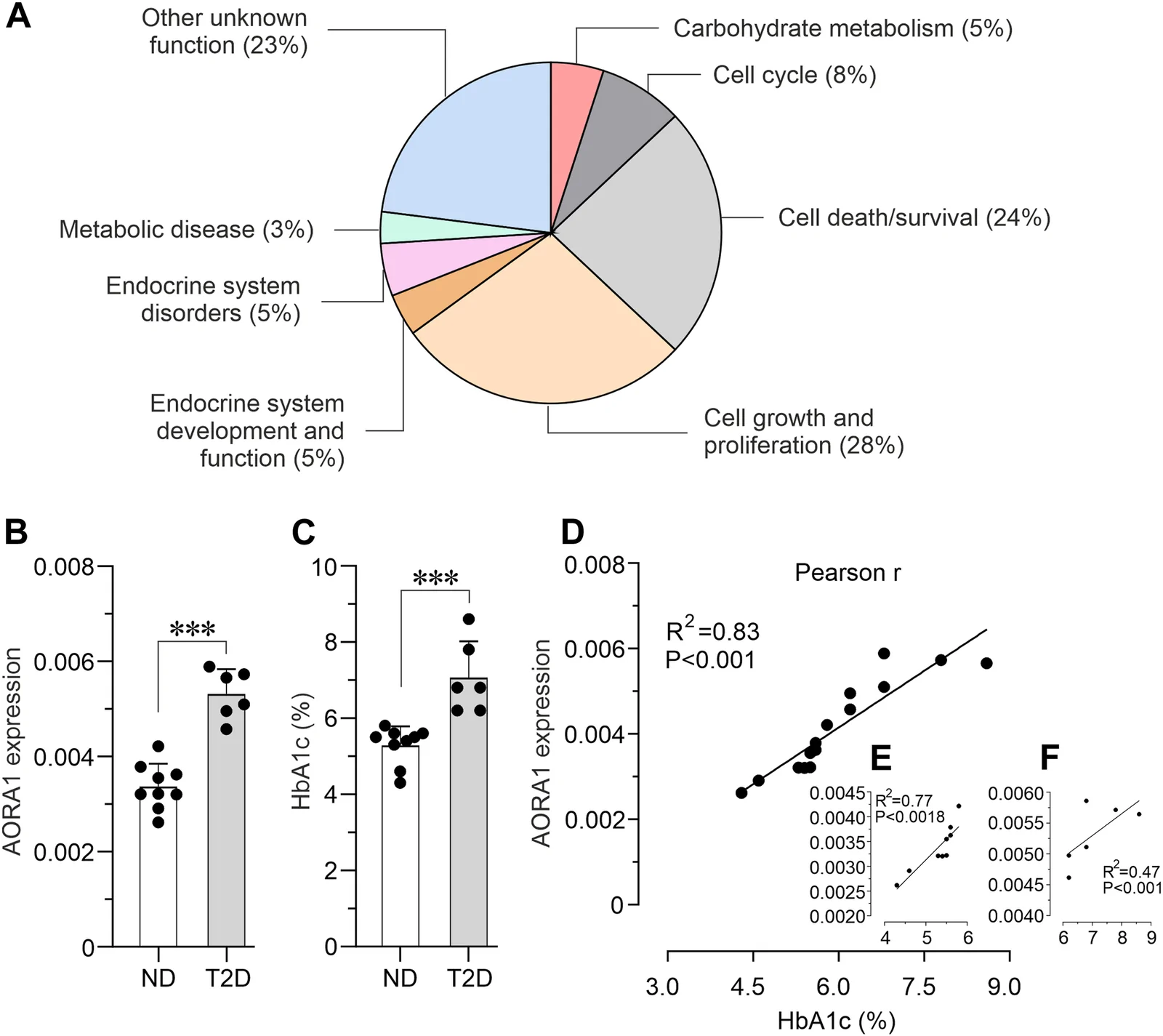
**Correlation of ADORA1 transcript expression with functionally important gene transcripts in human islets.***A*, functional annotations of 88 human islet genes strongly associated with ADORA1 mRNA expression were analyzed in RNA seq data (n = 89). *B*, increased expression of ADORA1 in ND (n = 9) and T2D (n = 6) islets analyzed. *C*, HbA1c levels in ND and T2D cadaveric donor subjects. *D*, positive correlation of ADORA1 mRNA expression analyzed by qPCR with HbA1c in pancreatic islets with insert indicting ADORA1 correlation with HbA1c in ND (n = 9) (*E*) or with T2D islets (n = 6) (*F*) separately. ∗∗*p* < 0.01 (unpaired *Student´;s t* test analysis).

Interestingly, qPCR analysis showed significantly higher ADORA1 mRNA expression in islets from donors with type 2 diabetes (T2D) compared with nondiabetic (ND) donors (Fig. 2, *B* and *C*). ADORA1 mRNA levels were strongly correlated with average blood glucose, measured as glycated hemoglobin (HbA1c), in the islets (Fig. 2*D*). When ND and T2D islets were analyzed separately, the correlation remained highly significant in both groups (Fig. 2, *E* and *F*, insert).

### Effect of adenosine on cytoplasmic Ca^2+^ and

Pancreatic β-cells secrete insulin in a pulsatile manner in response to glucose, driven by oscillations in cytosolic calcium ${\left[Ca^{2+}\right]}_{i}$ that are tightly coupled to glucose metabolism (31). The effect of adenosine (10 μM) on glucose-induced (20 mM) cytoplasmic Ca^2+^ oscillations was therefore examined. In the presence of adenosine, the oscillatory pattern of cytoplasmic Ca^2+^ in β-cells was rapidly and markedly suppressed and re-appeared after removal of adenosine (Fig. 3, *A* and *B*).

**Figure 3:**
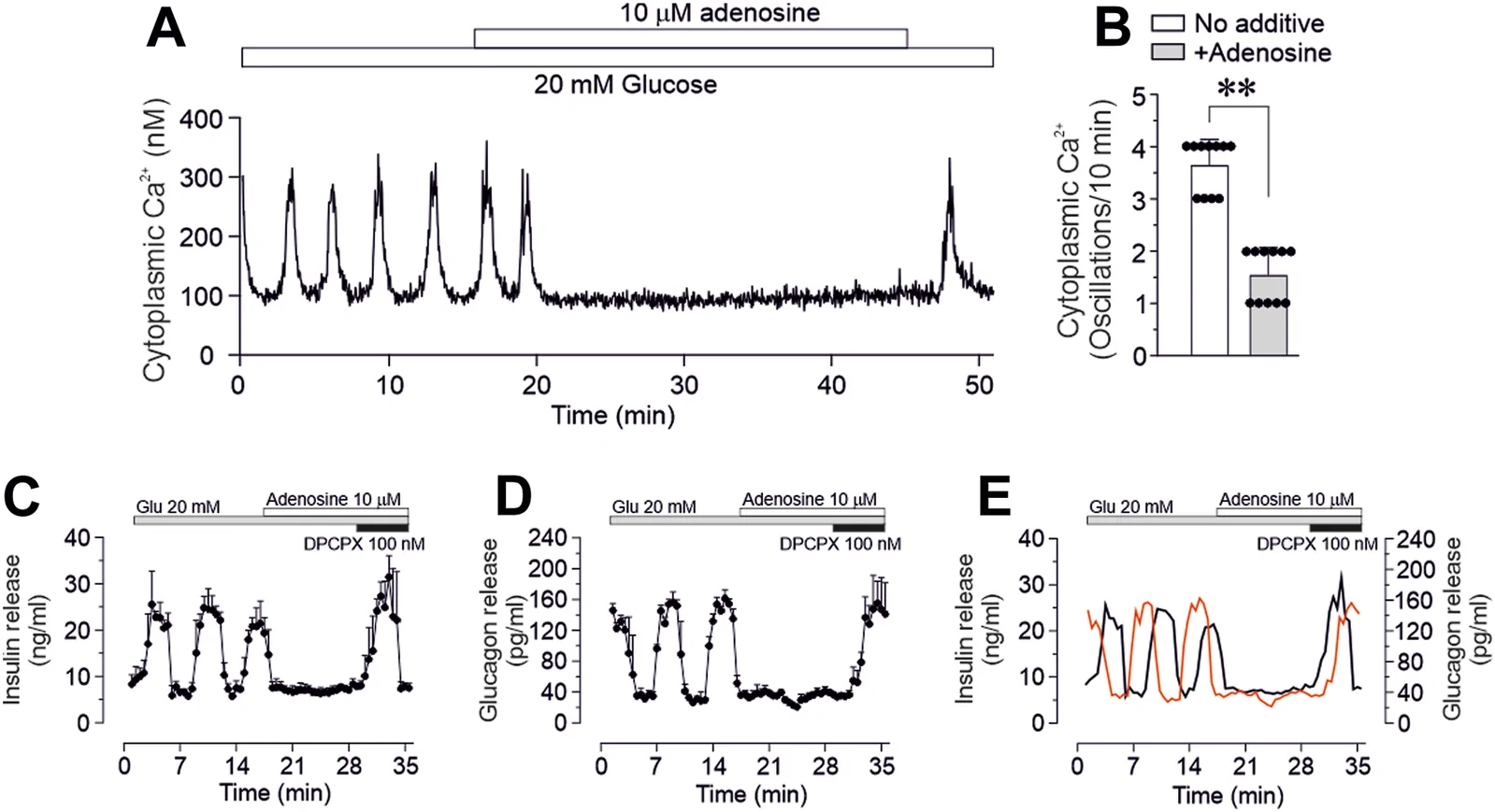
**Effect of adenosine on cytoplasmic Ca^2+^ oscillations in an individual β-cell and glucose-stimulated insulin secretion in isolated islets from *ob/ob* mouse.***A*, representative trace showing reversible disappearance of the oscillations during exposure to adenosine (10 μM). *B*, the frequency of the cytoplasmic Ca^2+^ oscillations as mean values ± SD for n = 11 observations in the presence and absence of adenosine. ∗∗*p* < 0.01 (unpaired *Student´s t* test analysis). (*C*) Isolated islets from *ob/ob* mouse were perfused with glucose (20 mM) revealing an oscillatory pattern of insulin (*C*) and glucagon (*D*) secretion. The temporal relationship insulin (*black line*) and glucagon secretion (*red line*) shown in (*E*). The secretory pattern of hormones was suppressed by addition of adenosine (10 μM) and re-appeared when DPCPX (100 nM) was added. Data are Mean ± SD from n = 6 different experiments.

### Effect of adenosine on pulsatile insulin and glucagon secretion in isolated ob/ob islets

Pancreatic β-cells from hyperinsulinemic *ob/ob* mice exhibit significantly elevated GSIS compared with β-cells from normoglycemic wild-type mice (32, 33). In subsequent experiments, the effect of adenosine on the dynamics of both insulin and glucagon secretion from perifused *ob/ob* and control mouse islets was evaluated.

As shown in Figure 3, perifusion of isolated islets from *ob/ob* mice with 20 mM glucose evoked a marked increase in insulin secretion characterized by rhythmic pulsatile fluctuations, with successive secretory peaks occurring at approximately 4-5-min intervals (Fig. 3*C*). Simultaneous measurement of glucagon in perifusate showed that elevation of glucose to 20 mM induced a rapid suppression of glucagon secretion, followed by partial recovery and oscillatory fluctuations (Fig. 3*D*). Superimposition of the insulin and glucagon secretory pattern revealed an inverse temporal relationship between the two hormones, with pulsatile increases in insulin generally coinciding with reduced glucagon secretion (Fig. 3*E*). Addition of adenosine (10 μM) to perifusion medium caused a rapid suppression of both hormones, an effect that was attenuated by subsequent addition of the selective ADORA1 antagonist DPCPX (100 nM) (Fig. 3, *C* and *D*).

In control C57BL/6 islets, raising glucose from 1 to 20 mM induced a biphasic insulin secretory response, characterized by a rapid first-phase peak followed by a weaker but sustained second phase (Fig. S1*A*). In contrast, glucagon secretion was rapidly and obviously suppressed following glucose elevation and remained substantially suppressed thereafter, with smaller oscillatory fluctuations (Fig. S1*B*). Similarly, addition of adenosine (10 μM) caused a rapid suppression of both insulin and glucagon, an effect that was attenuated by subsequent addition of DPCPX (100 nM) (Fig. S1, *A* and *B*). The inhibitory effect of glucose on glucagon secretion appeared more pronounced in control than in *ob/ob* islets (Figs. 3*D* and S1*B*). The temporal relationship between insulin and glucagon secretion in *ob/ob* and control islets is shown in Figures 3*E* and S1*C*. Notably, qPCR analysis showed that exaggerated GSIS in islets from *ob/ob* mice coincided with a reduced Adora1 mRNA expression as compared to C57BL/6 controls (Fig. S2, *A* and *B*).

### Insulin and glucagon secretion in isolated Adora1-KD islets

We next evaluated the dose-response relationship of glucose on insulin and glucagon secretion in isolated pancreatic islets from Adora1-knockdown (Adora1-KD) mice where the Adora1-KD efficiency was confirmed by qPCR (Fig. S2*C*). Islets were incubated for 60 min at glucose concentrations of 0, 2, 4, 8, 16, 20, 24, or 30 mM. In control islets, glucose stimulated insulin secretion starting at 8 mM, with a steeper increase at 16 mM and a plateau between 20 and 30 mM of glucose (Fig. 4*A*). This effect of glucose on insulin secretion was greatly increased at glucose concentrations above 16 mM in Adora1-KD mice (Fig. 4*A*).

**Figure 4:**
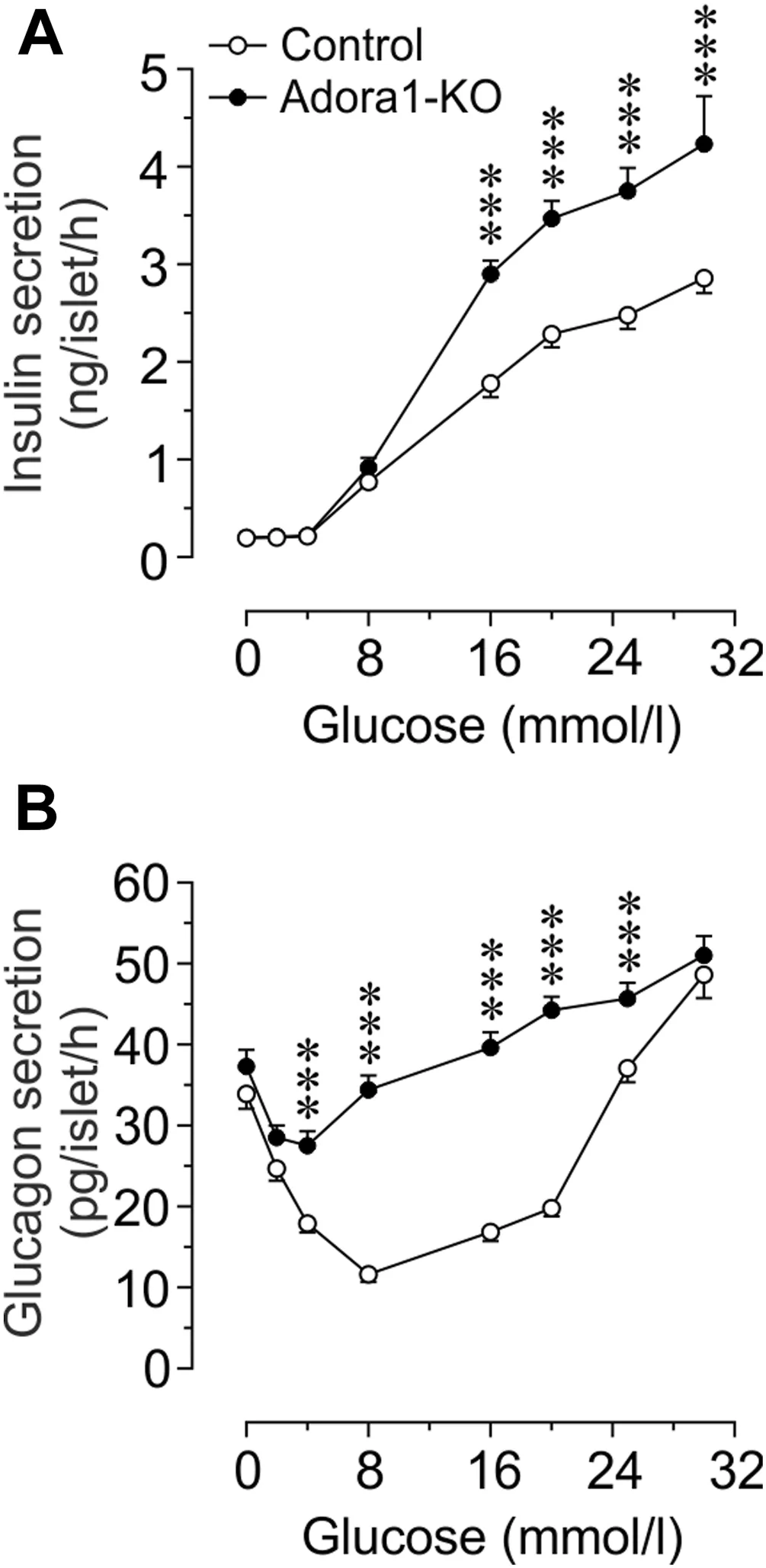
**Dose–response effect of glucose on insulin and glucagon secretion from isolated islets from control and Adora1-KD mice.** Isolated islets from control and Adora1-KD mice were incubated for 60 min and insulin (*A*) and glucagon (*B*) secretion in the absence (0) or presence of different glucose concentrations (2, 4, 8, 16, 20, 24, 30 mM) was measured. Mean ± SD for n = 6 mice in each group are shown. ∗∗∗*p* < 0.01 (unpaired *Student´s t* test analysis for each glucose concentration compared between control and AdoraA1-KD).

The suppressive effect of glucose on glucagon secretion that was already evident at 2 mM became more pronounced at 4 to 20 mM in control mice (Fig. 4*B*). At the highest glucose concentrations, however, the inhibitory effect on glucagon release was attenuated and glucagon secretion increased relative to lower glucose concentrations (Fig. 4*B*). Notably, the suppressive effect of glucose on glucagon secretion in the islets of Adora1-KD mice was greatly attenuated, which shows the significant role of Adora1 in the suppressive effect of glucose on glucagon secretion (Fig. 4*B*).

### Evaluation of insulin and glucagon secretion in Adora1-KD mice by pancreas perfusion

The effect of elevated glucose on insulin and glucagon secretion was further assessed by *in situ* pancreas perfusion in control and Adora1-KD mice. Analysis of insulin-data from individually perfused mouse in our previous study revealed that in control animals, an increase in glucose from 3.3 to 16.7 mM elicited a typical biphasic insulin secretory response, with a prominent first phase lasting 3 to 4 min followed by a flattened second phase that persisted for the duration of 16.7 mM glucose exposure (Fig. 5, *A*–*C*).

**Figure 5:**
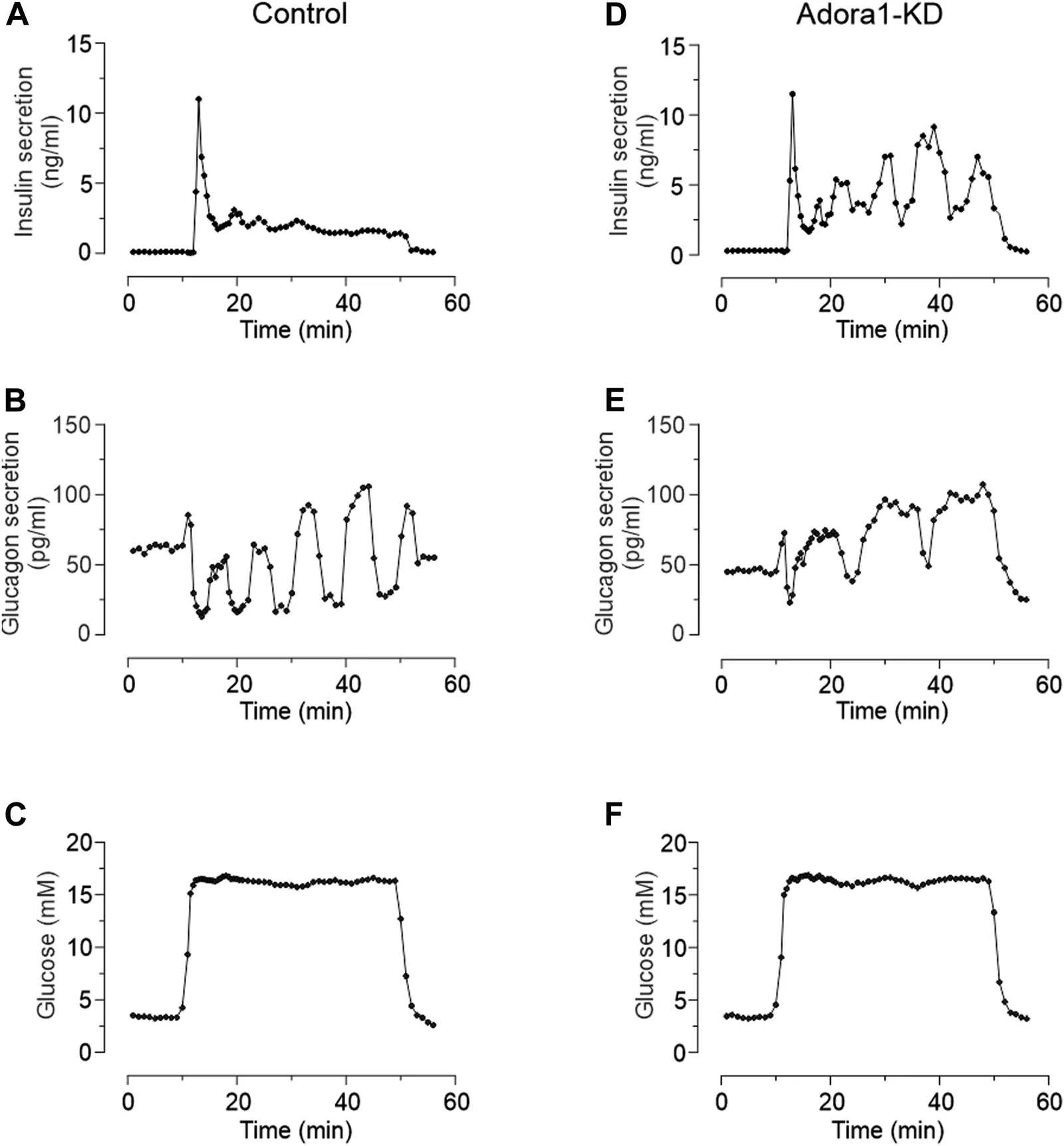
**Dynamics of insulin and glucagon secretion from perfused pancreas in control Adora1 (+/+) and Adora1 (−/−) mice.** Insulin (*A*) and glucagon (*B*) secretion measured by pancreas-perfusion before and after rise in glucose concentration from 3.3 to 16.7 mM (*C*) in control Adora1 (+/+) and Adora1 (−/−) mice. *D–F*, samples were taken at 60-s intervals, except during the first 10 min after increasing the glucose concentration (t = 11–21 min, when the sample interval was 30 s. A representative graph of the glucose level (*C* and *E*) in the same perfusate samples measured by the glucose oxidase method.

In Adora1-KD mice, the first phase of insulin secretion in response to the increase in glucose from 3.3 to 16.7 mM was comparably of the same magnitude as Adora1(+/+) mice (Fig. 5, *D*–*F*). In contrast, the second phase was markedly enhanced and displayed pronounced pulsatility (Fig. 5*D*).

The rise in glucose from 3.3 to 16.7 mM transiently increased glucagon release for approximately 1 min in control mice, followed by an inhibitory phase that was anti-synchronized with the first phase of insulin secretion (Fig. 5, *A*–*C*). During perfusion with 16.7 mM glucose, glucagon secretion exhibited repetitive secretory pulses from a baseline well below that observed at 3.3 mM glucose, with pulse half-widths of 4 to 5 min (Fig. 5, *B* and *C*). In Adora1-KD mice, increasing glucose from 3.3 to 16.7 mM similarly induced a brief rise in glucagon release, but, in contrast to Adora1(+/+), glucagon secretion was not suppressed below the baseline at 3.3 mM glucose (Fig. 5, *E* and *F*). During perfusion with 16.7 mM glucose, peak intervals and half-widths of glucagon pulses were prolonged to 8 to 10 min (Fig. 5, *E* and *F*). The typical insulin and glucagon response to increased glucose from 3.3 to 16.7 mM for individual mouse is shown in Fig. S3.

### Effect of Adora1-KD on high-fat diet–induced weight gain in mice

Because enhanced insulin secretion would be expected to promote nutrient uptake into peripheral tissues, the impact of a high calory diet, that is, high-fat diet (HFD) on body weight was investigated in control and Adora1-KD mice. A subset of animals was fed a HFD for 5 weeks. At the end of the treatment period, Adora1-KD mice exhibited significantly greater weight gain than control mice (Fig. 6, *A*–*D*).

**Figure 6:**
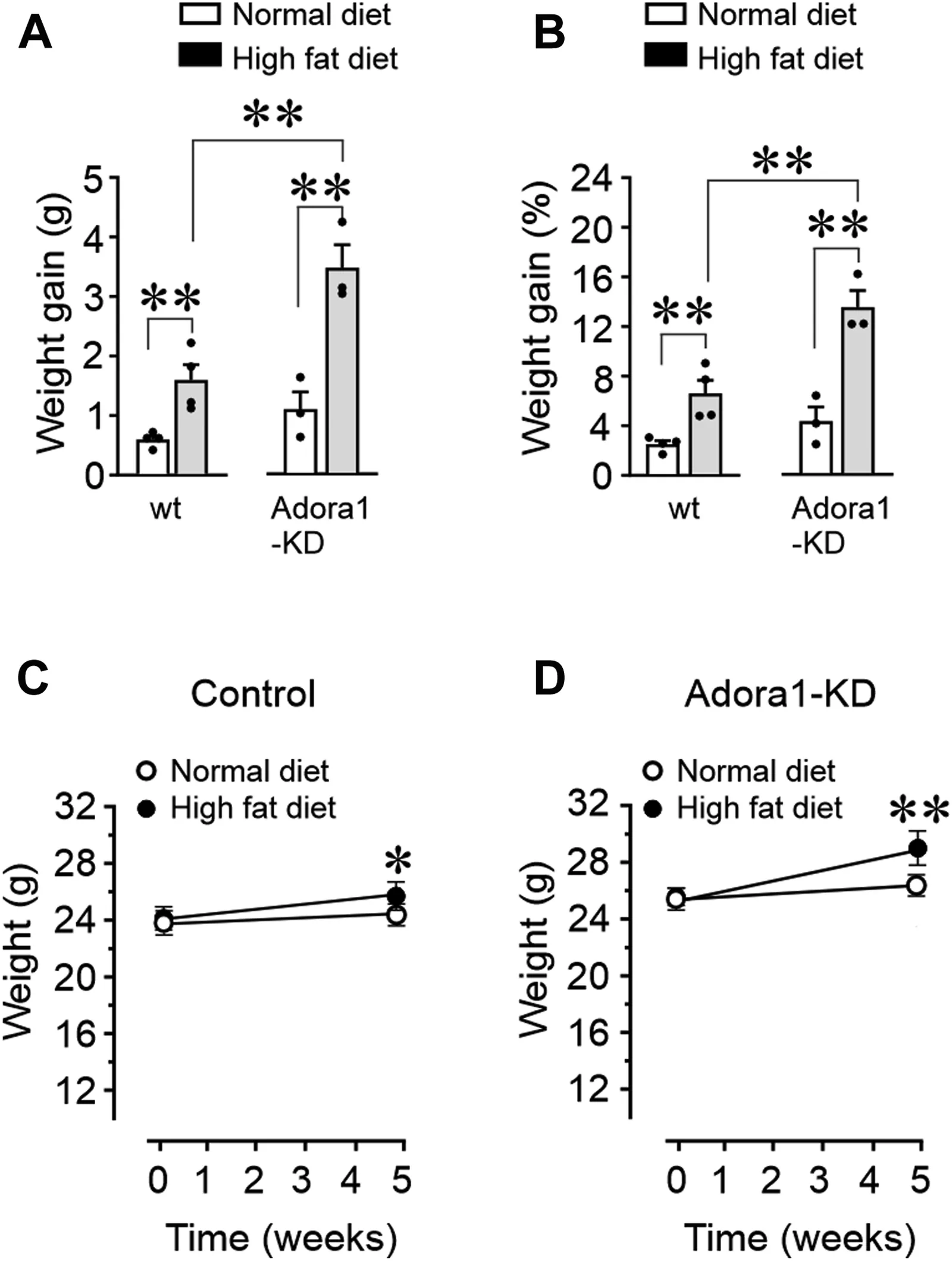
**Effect of high fat diet treatment in control and Adora1-KD mice.** Control and Adora1-KD mice were given a standard chow diet (normal diet) or a high fat diet for 5 weeks. Thereafter, the mice were weighed, and the body weight gain was calculated and presented as real values (weight in g) (*A* and *B*) and also as present of starting body weight (*C* and *D*). Mean ± SD for n = 4 mice in each group are shown. ∗*p* < 0.05; ∗∗*p* < 0.01 (Significance was assessed by unpaired *Student´s t* test between control and AdoraA1-KD).

### Effect of ADORA1 inhibition on glucose-stimulated insulin secretion in human islets

The potential effect of adenosine, in the presence or absence of the ADORA1 antagonist DPCPX, on insulin secretion was next assessed in isolated pancreatic islets from ND and T2D cadaveric donors. Islets were incubated at 1 or 16.7 mM glucose with adenosine (100 μM), DPCPX (1 μM), or both, and then washed, homogenized, and analyzed for cAMP content.

In ND islets, glucose-stimulated insulin secretion at 16.7 mM glucose was markedly suppressed by adenosine (Fig. 7*A*). DPCPX alone did not significantly affect GSIS but effectively prevented the inhibitory effect of adenosine (Fig. 7*A*). The effects of adenosine and DPCPX on insulin secretion were mirrored in the cAMP content of islets (Fig. 7*B*).

**Figure 7:**
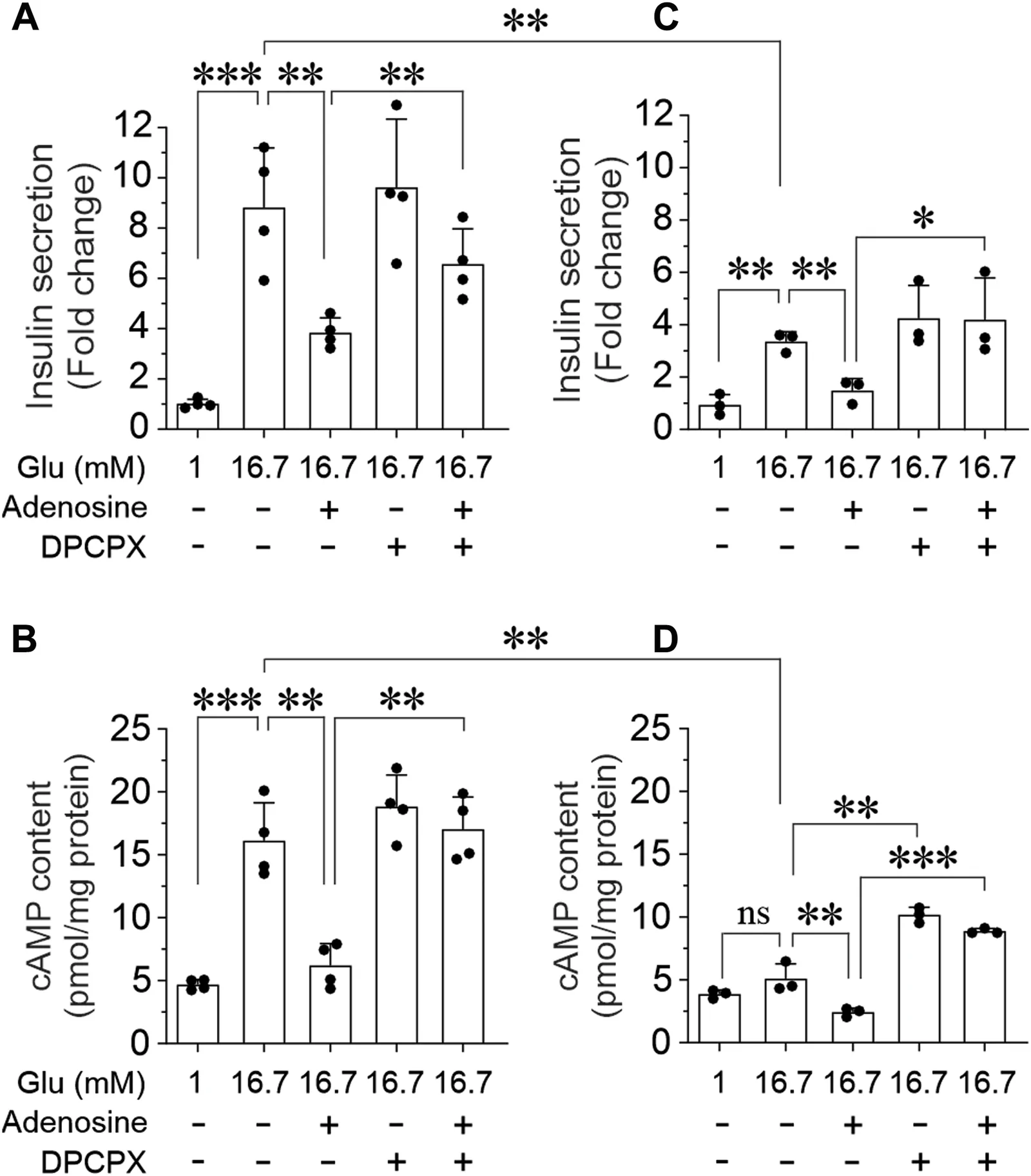
**Effect of Adora1 inhibitor on adenosine-induced suppression of insulin release in islets from ND and T2D donors.** Isolated human islets were incubated for 60 min at 1 or 16.7 mM glucose in the presence of adenosine (100 nM), DPCPX (1 uM) or adenosine + DPCPX. Thereafter the islets were washed with PBS and sonicated whereby cAMP was measured in islet lysates. *A*, glucose-stimulated insulin secretion and cAMP generation (*B*) were suppressed by adenosine in ND islets. DPCPX did not affect insulin secretion or cAMP generation at 16.7 mM glucose, but it counteracted the suppressive effect of adenosine on glucose-stimulated insulin secretion and cAMP generation in ND islets. Glucose-stimulated insulin secretion and cAMP level was significantly lower in incubated T2D islets at 16.7 mM glucose compared to ND islets (*C* and *D*). Adenosine induced further suppression of both glucose-stimulated insulin secretion and cAMP generation. DPCPX counteracted the suppressive effects of adenosine on glucose-stimulated insulin secretion and cAMP generation. The experiments were performed at different occasions with islets from ND cadaveric donors (n = 4) and T2D cadaveric donors (n = 3). Significance was assessed by unpaired *Student´s t* test between compared groups ∗ *p* < 0.05; ∗∗*p* < 0.01, ∗∗∗*p* < 0.001.

In T2D islets, GSIS was significantly reduced compared to ND islets (*p* < 0.05), and this modest response of T2D islets was further suppressed by adenosine (Fig. 7*C*). DPCPX counteracted the inhibitory effect of adenosine on GSIS (Fig. 7*C*). Unlike ND islets, 16.7 mM glucose did not significantly increase cAMP levels in T2D islets, although adenosine further reduced cAMP (Fig. 7*D*). DPCPX reversed the adenosine-induced suppression of cAMP levels in T2D islets (Fig. 7*D*).

### Beneficial effect of DPCPX on β-cell reductive capacity in human islets

The impact of ADORA1 activation by adenosine on β-cell reductive capacity, assessed as formazan formation (an index of mitochondrial reductive capacity and an indirect measure of cell viability), (34, 35), was then investigated. Human islets from ND or T2D donors were cultured for 72 h at basal (5 mM) or high (20 mM) glucose in the presence or absence of adenosine (100 μM) or DPCPX (1 μM).

In ND islets, high glucose (20 mM) reduced reductive capacity compared with 5 mM glucose, and the presence of adenosine further exacerbated this glucotoxic effect (Fig. 8*A*). DPCPX counteracted the detrimental effects of both high glucose and adenosine, restoring reductive capacity to levels comparable to those observed at 5 mM glucose (Fig. 8*A*). In T2D islets, cellular viability was already reduced at basal glucose (5 mM) and declined further at 20 mM glucose, with an additional decrease in the presence of adenosine (Fig. 8*B*). As in ND islets, DPCPX reversed the negative impact of high glucose and adenosine on the reductive capacity in T2D islets (Fig. 8*B*).

**Figure 8:**
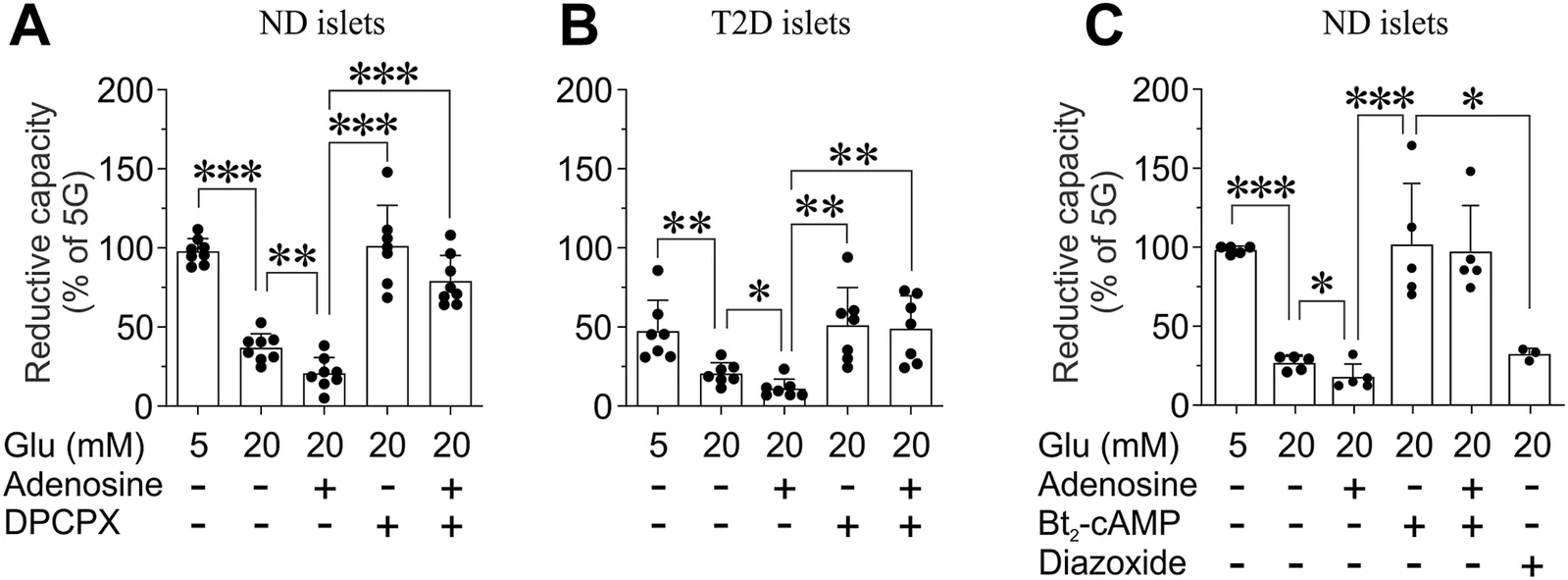
**Effect adenosine on cell viability in cultured human islets.** Effects on cell viability (measured by MTS) after treatment with adenosine (100 μM), DPCPX (1 μM) or both on isolated human islets from non-diabetic cadaveric donors (ND islets) (*A*) or type 2 diabetic cadaveric donors (T2D islets) (*B*) cultured at 20 mM glucose for 72 h. Cell viability in ND islets cultured at high glucose (20 mM) with adenosine (100 μM), Bt_2_-cAMP (100 μM) or diazoxide (200 μM) for 72 h shown in (*C*). Control islets cultured at basal glucose (5 mM) in all groups are included. Means ± SD for n = 5 to 8 ND observations and n = 7 T2D observations are shown. Differences between groups were analyzed using an unpaired Student's *t* test. ∗*p* < 0.05; ∗∗*p* < 0.01; ∗∗∗*p* < 0.001.

Because reduced cAMP generation is a major consequence of adenosine signaling, the effect of a cell-permeable cAMP analogue, dibutyryl cAMP (Bt_2_-cAMP, 100 μM), was examined in ND human islets cultured at 20 mM glucose with or without adenosine (100 μM). For comparison, the potential protective effect of diazoxide (200 μM), a K^+^_ATP_ channel opener reported to induce “β-cell rest” and reduce apoptosis, was also tested under high-glucose conditions. Bt_2_-cAMP efficiently counteracted the decreased reductive capacity induced by high glucose and adenosine, restoring that to near-control levels observed at 5 mM glucose (Fig. 8*C*). In contrast, diazoxide did not exert any detectable protective effect on the reductive capacity under glucotoxic conditions (Fig. 8*C*).

Since the MTS assay mainly reflects cellular metabolic activity and reductive capacity rather than direct cell viability (29), two complementary assays were employed to determine whether the observed effects of adenosine were associated with changes in cell viability and apoptosis. Cell viability was assessed using the Calcein AM assay, whereas apoptosis was quantified by measuring cytoplasmic histone-associated DNA fragments (mono- and oligonucleosomes), which is a well-established marker of apoptosis, using a Cell Death Detection ELISA kit (29).

As shown in Figure 9*A*, adenosine reduced INS-1832/13 cell viability in a concentration-dependent manner under basal glucose (5 mM), reaching statistical significance at 10 μM. In contrast, neither the ADORA1 receptor antagonist DPCPX (1 μM) nor Bt_2_-cAMP (100 μM) significantly affected cell viability under basal glucose conditions (Fig. 9*A*). Exposure of INS-1832/13 cells to high glucose (20 mM) for 72 h, however, markedly reduced cell viability, and this reduction was further enhanced by adenosine (10 μM) (Fig. 9*B*). Both DPCPX and Bt_2_-cAMP significantly prevented the decrease in cell viability induced by high glucose alone and by the combination of high glucose and adenosine (10 μM) (Fig. 9*B*).

**Figure 9:**
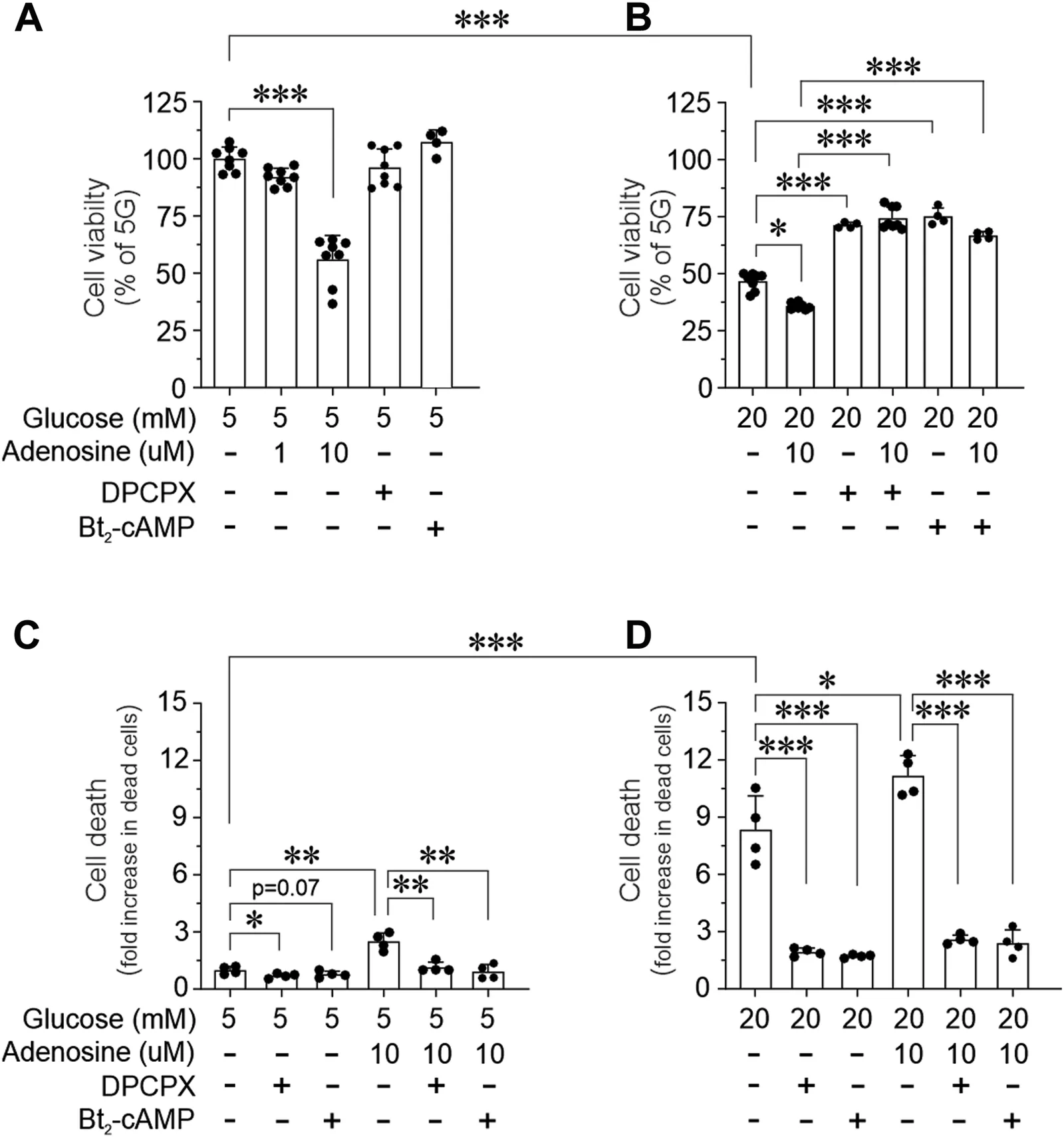
**Effects of adenosine on β-cell viability and apoptosis.** Cell viability was assessed using the Calcein AM, fluorescence-based viability assay, after culturing INS-1832/13 cells for 72 h at basal (5 mM) or high (20 mM) glucose in the presence or absence of the indicated agents. *A*, under basal glucose (5 mM) conditions, adenosine (1 and10 μM) significantly reduced cell viability at 10 μM. *B*, high glucose decreased cell viability, and this effect was further enhanced by adenosine (10 μM). DPCPX (1 μM) and Bt_2_-cAMP (100 μM) affecting cell viability at 5 mM glucose, significantly attenuated the reduction in cell viability induced by high glucose alone or in combination with adenosine. *C–D*, apoptosis was quantified using a Cell Death ELISA by measuring cytoplasmic histone-associated DNA fragments (nucleosomes). Under basal glucose conditions (5 mM), adenosine (10 μM) increased apoptosis, whereas DPCPX (1 μM) and Bt_2_-cAMP (100 μM) prevented this effect. High glucose (20 mM) markedly increased apoptosis, which was further enhanced by adenosine (10 μM), while DPCPX (1 μM) and Bt_2_-cAMP (100 μM) significantly attenuated apoptosis induced by high glucose alone or together with adenosine. Data are mean ± SD of n = 4 to 8 independent experiments. The significance between two groups was evaluated by an unpaired Student's *t* test. ∗*p* < 0.05; ∗∗*p* < 0.01; ∗∗∗*p* < 0.001.

To determine whether the reduction in cell viability was associated with apoptotic cell death, apoptosis was assessed (29). Under basal glucose conditions, DPCPX (1 μM) significantly reduced basal apoptosis and effectively prevented the increase in apoptosis induced by adenosine (10 μM) (Fig. 9*C*). Bt_2_-cAMP (100 μM) produced a similar protective effect against adenosine-induced apoptosis, although it did not significantly alter basal apoptosis (Fig. 9, *C* and *D*). Exposure of INS-1832/13 cells to high glucose (20 mM) for 72 h markedly increased apoptosis, which was further exacerbated by adenosine (10 μM) (Fig. 9*D*). Importantly, both DPCPX and Bt_2_-cAMP significantly attenuated apoptosis induced by high glucose alone as well as by the combined treatment with high glucose and adenosine (Fig. 9*D*).

### Suppression of β-cell proliferation by adenosine and reversal by DPCPX

The effect of adenosine on proliferation of INS-1832/13 cells was evaluated using a [^3^H]thymidine incorporation assay, a well-established method for quantifying cell proliferation. INS-1832/13 cells cultured at 11.2 mM glucose displayed a basal proliferation rate that was significantly suppressed by adenosine at all concentrations tested (1, 10, and 100 μM) (Fig. S3, *A* and *B*). DPCPX (1, 10, or 100 μM) did not affect basal proliferation but prevented the adenosine-induced reduction in INS-1832/13 cell proliferation (Fig. S4, *A* and *B*).

## Discussion

The present findings position ADORA1 as a central modulator of islet hormone secretion, β-cell survival during pathophysiological conditions, and also impacting the proliferative capacity of β-cells under diabetic conditions, with strong support for ADORA1 inhibition as a therapeutic strategy to preserve or restore β-cell function in type 2 diabetes (T2D).

### ADORA1 in the pathophysiology of T2D

T2D is characterized by a combination of insulin resistance and progressive β-cell dysfunction, driven in large part by chronic glucotoxicity and glucolipotoxicity (3, 19, 29, 36). The present data show that ADORA1 expression is increased in human islets from donors with T2D and that ADORA1 mRNA correlates with HbA1c, directly linking this receptor to the glycemic burden and β-cell failure in human disease. This is consistent with the broader concept that stress-related signaling pathways are upregulated in islets as diabetes progresses (37, 38, 39, 40), but here ADORA1 emerges not only as a marker of disease severity but as an active mediator of β-cell dysfunction.

The observation that ADORA1 inhibition (by DPCPX or genetic knockdown) improves insulin secretory dynamics, preserves β-cell viability under glucotoxic conditions, and supports β-cell proliferation addresses several key pathophysiological components of T2D simultaneously such as impaired GSIS, dysregulated glucagon secretion, and progressive loss of β-cell mass. Together, the data suggest that maladaptive activation of ADORA1 in the diabetic islet contributes to both functional and structural β-cell deterioration, and that selective ADORA1 antagonism has the potential to counteract these processes.

### Integration with prior work on adenosine and β-cell function

Adenosine has long been implicated in the discussion of glucose homeostasis (9, 11, 13, 16, 18), but its actions have been complex and sometimes contradictory, reflecting concentration-dependent effects and the involvement of multiple receptor subtypes (ADORA1, ADORA2A, ADORA2B, ADORA3) in whole-body context (15, 18). Earlier studies in rodent islets reported that micromolar concentrations of adenosine suppress insulin secretion, whereas higher (millimolar) concentrations can enhance insulin release, indicating that receptor context and signaling balance determine the net effect (9, 13). The present data refine this framework in human and mouse islets by demonstrating that ADORA1 and ADORA2A are the predominant adenosine receptor transcripts, whereas ADORA2B and ADORA3 are expressed at very low or trace levels, and by functionally linking ADORA1 activation to suppression of β-cell Ca^2+^ oscillations, insulin pulsatility, and cell viability.

Importantly, prior *in vivo* work has shown that non-selective adenosine receptor antagonists (*e.g*. aminophylline, pentoxifylline) can enhance insulin secretion or improve insulin sensitivity by modulating adenosine signaling in multiple tissues, including muscle and adipose tissue (11, 18). However, these systemic approaches did not resolve the specific role of islet ADORA1, nor did they address the paradox that adenosine signaling has been reported to promote β-cell regeneration in some experimental systems (41). Andersson and colleagues demonstrated that NECA, a non-selective adenosine receptor agonist, stimulates β-cell proliferation and lowers blood glucose in zebrafish and rodent models, suggesting that adenosine signaling can drive β-cell expansion in certain contexts (41). The present work provides an apparent reconciliation of these findings by showing that, under chronic high-glucose stress, ADORA1 signaling in mammalian β-cells is predominantly detrimental as it suppresses insulin secretion, reduces β-cell proliferation, and exacerbates glucotoxic cell damage, whereas antagonism of ADORA1 reverses these effects.

Taken together, the current data support a model in which adenosine signaling is beneficial or regenerative when it is routed through other receptor subtypes and under defined experimental conditions but becomes maladaptive when chronic hyperglycemia preferentially engages ADORA1 in human and mouse islets. This suggestion is crucial for therapeutic development, because it suggests that receptor-selective targeting (favoring ADORA1 blockade while preserving or redirecting signaling through other adenosine receptors) may allow exploitation of the beneficial aspects of adenosine biology without aggravating β-cell dysfunction.

### ADORA1 control of islet Ca^2+^ dynamics and hormone pulsatility

Insulin and glucagon are normally secreted in a pulsatile manner, and the integrity of these secretory oscillations is critical for maintaining peripheral insulin sensitivity and hepatic glucose output (5, 12, 18, 42). The present data demonstrate that adenosine, at concentrations relevant for receptor activation, rapidly suppresses the glucose-induced cytoplasmic Ca^2+^ oscillations in β-cells, thereby dampening pulsatile insulin secretion in perifused mouse islets. In parallel, adenosine also abolishes pulsatile glucagon secretion from mouse islets (*cf.* Figs. 3 and S1 for the impact on insulin and glucagon secretion). Both these effects are reversed by DPCPX, pointing to ADORA1 as the key mediator of adenosine-induced disruption of insulin and glucagon pulsatility in both *ob/ob* and control C57BL/6 mouse islets.

These findings integrate well with the known electrophysiological role of ADORA1 (9, 18). ADORA1 is canonically coupled to G_i/o_ proteins and can activate K^+^_ATP_ channels, leading to membrane hyperpolarization and suppression of exocytosis (9, 16, 18). Thus, the loss of Ca^2+^ oscillations and the blunting of insulin and glucagon pulses observed here are mechanistically consistent with classical ADORA1 signaling and provide direct functional evidence in intact islets (12). Furthermore, the demonstration that Adora1-KD in mice preserves and even enhances second-phase, pulsatile insulin secretion during pancreas perfusion suggests that tonic ADORA1 activation acts as a brake on β-cell excitability and that removing this brake can restore more robust and physiologically appropriate secretion.

Considering the disrupted insulin pulsatility and inappropriate glucagon secretion as hallmarks of T2D (5, 43), the ability of ADORA1 antagonism to normalize these dynamic features of islet hormone release places ADORA1 receptor at the intersection of key pathophysiological defects. This adds to prior work (19) showing that, in addition to the intracellularly generated signals, paracrine and intrinsic GPCR signaling is undoubtedly also important for β- and α-cells oscillatory behavior and supports the notion that targeting ADORA1 can reestablish normal secretory patterns in diabetes.

### ADORA1, glucotoxicity, and β-cell survival

Chronic exposure to high glucose (and combined glucolipotoxicity) leads to β-cell dedifferentiation, functional exhaustion, and apoptosis, contributing irreversibly to β-cell mass loss in T2D (2, 29, 36, 44, 45). The current data show that adenosine, *via* ADORA1, aggravates the deleterious effects of high glucose on β-cell reductive capacity in human islets from both ND and T2D donors, whereas ADORA1 blocker DPCPX almost completely restores cell viability to basal levels. The suppressive effect of adenosine on cellular reductive capacity was accompanied by reduced cell viability and increased apoptosis, indicating that adenosine affects both cellular metabolism and survival. This strongly implicates ADORA1 as a critical amplifier of glucotoxic stress in islet β-cells. Thus, these observations are consistent with broader literature implicating cAMP and protein Kinase A (PKA) system as of central importance for β-cell survival under metabolic stress (34, 44, 46, 47, 48). Generation of cAMP in β-cells promotes pro-survival signaling, enhances GSIS, and supports cellular function, whereas reductions in cAMP are associated with increased susceptibility to glucotoxic and glucolipotoxic damage (34, 46, 47, 48). The finding that adenosine reduces cAMP in ND and T2D islets and that DPCPX reverses this effect aligns with the canonical G_i_-coupling of ADORA1 and provides a mechanistic link between ADORA1 activation, cAMP depletion, and impaired β-cell survival.

The rescue of cell viability by Bt_2_-cAMP, even in the combined presence of high glucose and adenosine, further supports cAMP as a key downstream mediator of ADORA1 signaling in β-cells and positions ADORA1 upstream of a critical survival pathway. In contrast, diazoxide, a K^+^_ATP_ channel opener used to induce “β-cell rest” (49, 50), failed to confer significant protection in this model, suggesting that under glucotoxic conditions, negatively modulated cAMP signaling *via* ADORA1 may be more impactful than purely electrophysiological interventions (49, 50). This is in line with recent work emphasizing cAMP and its upstream-mediated signaling mechanisms in preventing β-cell death in T2D, beyond mere over-stimulation by elevated glucose (19, 34, 39, 44, 48, 51).

### ADORA1 and β-cell proliferation

Loss of β-cell mass in T2D reflects an imbalance between β-cell death and insufficient regenerative responses (29, 48, 52). While some studies have suggested that adenosine signaling can promote β-cell proliferation in development or regenerative models (41), the current data show that, in INS-1832/13 β-cells under standard glucose conditions, adenosine consistently suppresses cell viability and proliferation at all concentrations tested, and that this anti-proliferative effect is reversed by DPCPX. These results indicate that, at least in this mammalian β-cell context, ADORA1 activation inhibits β-cell proliferation, while its blockade permits maintenance of normal proliferative capacity. This apparently contrasts with NECA-induced β-cell regeneration reported in earlier *in vivo* studies (41), but the discrepancy can be resolved by considering receptor subtype specificity and model differences. NECA is a non-selective agonist that can activate ADORA2A and ADORA2B, which are positively coupled to cAMP (15, 18) and have been implicated in pro-regenerative responses, whereas the INS-1832/13 model in the current study isolates the inhibitory, G_i_-coupled target of adenosine signaling *via* ADORA1. Thus, the present findings do not contradict the concept of adenosine-driven β-cell regeneration; instead, they indicate that regenerative signaling likely requires activation of non-ADORA1 receptors (18, 41), and that ADORA1 activation in β-cells is predominantly anti-proliferative.

From a therapeutic standpoint, this suggests that selective ADORA1 antagonism could be combined with strategies that enhance signaling through pro-regenerative adenosine receptors, potentially allowing simultaneous protection against glucotoxic injury and promotion of β-cell mass growth. Such a dual approach might be particularly beneficial and desirable in early or preclinical T2D, when residual regenerative capacity remains.

### Islet ADORA1, glucagon dysregulation, and systemic metabolism

Dysregulated glucagon secretion, characterized by fasting hyperglucagonemia and impaired suppression by glucose, is a major driver of increased hepatic glucose production in T2D (43, 53, 54, 55). The present data show that ADORA1 is expressed not only in β-cells but also in α-cells in both human and mouse islets and that adenosine exposure suppresses pulsatile glucagon secretion in mouse islets, an effect reversed by DPCPX. Moreover, genetic knockdown of Adora1 alters the pattern and temporal structure of glucagon pulses during pancreas perfusion, indicating that ADORA1 contributes to the finetuning of α-cell activity and its coupling to β-cell secretion.

The current findings complement previous studies (12, 18) suggesting that adenosine signaling *via* ADORA1 in α-cells modulates glucagon release and may be altered in diabetes, where changes in ADORA1 expression have been linked to hyperglucagonemia in certain settings. In the present study, the predominant finding is suppressive, but the key translational insight is that ADORA1 constitutes a shared molecular node in both β- and α-cells. Insulin is the most predominant and potent suppressor of glucagon secretion and exerts its effect through a combination of both direct and indirect mechanisms (25, 56, 57, 58). Thus, negatively modulating ADORA1 activity, with the result of improved GSIS, could therefore reestablish the appropriate reciprocal relationship between insulin and glucagon secretion where insulin suppresses glucagon secretion, which is essential for restoring physiological glucose homeostasis.

The increased weight gain observed in Adora1-KD mice on a high-fat diet, despite improved insulin secretory dynamics, underscores the complexity of systemic ADORA1 biology. ADORA1 in adipose tissue and muscle promotes insulin sensitivity and limits lipolysis, and global ADORA1 inhibition has been associated with increased circulating lipids and altered substrate utilization (14, 18). Thus, while islet-specific ADORA1 inhibition appears clearly beneficial for β-cell function and survival, whole-body ADORA1 blockade might have less favorable effects on lipid metabolism and weight, emphasizing the need for tissue-selective targeting strategies or delivery approaches that focus on the islets. However, it is not surprising that improved insulin secretion *via* ADORA1 inhibition and the anti-lipolytic effect of insulin would have beneficial effects and could counteract dysregulation of blood lipids.

### Therapeutic implications: ADORA1 as a drug target in T2D

Taken together, the present data show that ADORA1 is (i) highly expressed in human islets, (ii) correlated with glycemic burden in the islets and upregulated in T2D islets, and (iii) functionally involved in suppressing insulin secretion, disrupting hormone pulsatility, reducing β-cell survival under glucotoxic stress, and inhibiting β-cell viability and proliferation. ADORA1 blockade by DPCPX or genetic knockdown reverses these defects, normalizes or enhances GSIS, restores β-cell viability *via* preserving cAMP signaling, and allows maintenance of proliferative capacity.

Within the broader framework of adenosine biology in metabolism, this positions ADORA1 as an attractive and mechanistically well-defined target for β-cell-directed therapies in T2D. In contrast to current incretin-based or insulin-sensitizing drugs, which primarily enhance existing β-cell function in a β-cell-stressing manner, or reducing insulin demand in an improper manner (53, 59), ADORA1 inhibition directly addresses intrinsic β-cell stress signaling and survival pathways. Furthermore, because ADORA1 also modulates α-cell secretion, selective antagonism has the potential to simultaneously correct both insulin and glucagon abnormalities, offering a more integrated approach to restoring islet function.

Future work should address the development of β-cell-selective ADORA1 antagonists, possibly *via* targeted delivery systems or biased ligands, and evaluate their efficacy and safety in preclinical models of T2D with intact immune and metabolic axes. Considering the complex systemic roles of ADORA1, including beneficial effects on insulin sensitivity in muscle and adipose tissue (14, 18), an ideal therapeutic strategy may involve partial or tissue-restricted inhibition rather than complete systemic blockade. Nonetheless, the present data provide strong experimental support for ADORA1 as a key mediator of β-cell dysfunction in T2D and highlight ADORA1 antagonism as a promising avenue for disease-modifying treatment focused on preserving and restoring pancreatic β-cell function.

## Experimental procedure

### Animal

The experiments were performed both with obese-hyperglycemic (*ob/ob*) male mice (16–18 weeks of age) taken from a local colony (Umeå, Sweden) and male mice of C57/BL/6 background expressing Adora1 (+/+) or Adora1 (−/−) (16–18 weeks of age). The Adora1 (−/−) mice (B6N.129P2-Adora1tm1Bbf/J) were back-crossed to a C57/BL/6 congenic strain by Jackson Laboratory according to the general procedures for back-crossing until the mice were determined to be congenic by 140 genomic markers. Adora1 (+/+) and Adora1 (−/−) mice were bred from Adora1 (+/−) parents, and PCR-based genotyping was used to identify the Adora1 (−/−) offspring as reported previously (60). The mice were allowed free access to standard chow diet containing 10 kcal% or high fat diet with 60 kcal% fat, respectively (Research Diets). The experimental protocols were approved by local ethical committees.

### Chemicals

Reagents of analytical grade and deionized water were used. Adenosine, 8-cyclo-pentyl-1,3-dipropylxanthine (DPCPX) and adenosine were obtained from Sigma-Aldrich (St Louis, MO). Roche Diagnostics supplied collagenase, bovine serum albumin and HEPES. Fatty acid-free bovine serum albumin, insulin ELISA kit (Mercordia), Primers (Table S1), and qPCR materials was from Applied Biosystems. The acetoxymethyl ester of Fura 2 was purchased from Molecular Probes. All other chemicals were from Merck AG or Sigma.

### Confocal microscopy

Isolated human or mouse pancreatic islets were fixed with 4% formaldehyde, permeabilized with 5% Triton X-100 and unspecific bindings were blocked with 5% Normal Donkey Serum (Jackson ImmunoResearch Laboratories Inc). The expression of ADORA1 was detected with rabbit anti-ADORA1 (1:200) (ab, 3460) in combination with Cy3-conjugated anti-rabbit IgG (1:100) (Jackson Immunoresearch Laboratories Inc). For staining of insulin, we used guinea pig-raised anti-insulin antibody (1:400) (Eurodiagnostica, Malmö, Sweden) followed by incubation with a Cy5-conjugated anti-guinea pig IgG antibody (1:150). The fluorescence was visualized with a Zeiss LSM510 confocal microscope by sequentially scanning at (excitation/emission) 543/570(Cy3) and 633/>650 nm (Cy5), as has also been described elsewhere (29, 46).

### Measurements of cytoplasmic Ca^2+^ oscillations in isolated β-cells

Detection of cytoplasmic Ca^2+^ and its oscillations in isolated β-cells is described in supplementary materials as previously described (61, 62, 63).

### Measurements of hormone release from the perfused pancreas

*In situ* perfusion of the pancreas was performed as previously described (42, 64, 65), and the medium concentrations of insulin (39) and glucagon (66, 67) were analyzed by radioimmunoassay (see supplementary materials).

### Insulin and glucagon secretion from isolated rodent islets

Freshly isolated islets were preincubated for 30 min at 37 °C in Krebs-Ringer bicarbonate buffer (KRB), pH7.4, supplemented with 10 mM HEPES, 0.1% BSA, and 1 mM glucose as previously described (46). Each incubation vial, containing 10 islets/ml medium, was gassed with 95% O_2_ and 5% CO_2_ to obtain a stable and constant pH and oxygenation. After preincubation, the buffer was changed to a medium containing glucose and test agents as specified, and the islets in each vial were incubated in a volume of 1 ml at 37 °C in a metabolic shaker (30 cycles/min). Thereafter, aliquots of the medium were removed for the assay of insulin and glucagon as previously described (26, 46). For the perifusion of isolated islets, 50 islets were transferred to a perifusion column, where they were sandwiched between two layers of gel (Bio-gel P-4, 200–400 mesh) (Bio-Rad Laboratory) and perifused at a rate of 0.25 ml/min with the KRB buffer (68). The perifusion medium was gassed with 95% O_2_ and 5% CO_2_ and glucose (20 mM) and the test substances were introduced following the initial exposure to 1 mM glucose for 4 to 5 min, according to the protocols (Figs. 3 and S1). The insulin and glucoagon levels in the perfusates were determined with a radioimmunoassay kits (69).

### Insulin secretion from isolated human islets

The isolated human islets were incubated in KRB buffer in a similar manner to mouse islets described earlier. A more detailed description could be found in supplementary materials.

### cAMP determination

After incubation of islets, they were washed and removed to a new tube and treated with 100 mM HCl for 5 min and then frozen. On the day of analysis samples were sonicated on ice and cAMP was measured using a cAMP ELISA kit (Enzo Life Sciences) according to the manufacturer’s instruction and the values were related to protein content of each sample. The protein concentrations of the cell lysates were measured by a BCA kit (Thermo Fisher Scientific) as reported previously (34, 46).

### Cell viability assay (MTS and Calcein AM)

After culture of islets at 5 mM or a high glucose concentration (20 mM) in the absence or presence of different test-agents the islets were dispersed into single cells using Ca^2+^ free-medium. Measurement of cell viability was performed using either MTS (Promega) and Calcein AM (Thermo Fisher Scientific) reagents according to manufacturer’s instructions.

### Sample size

Sample size determination was performed by *a priori* power analysis using G∗Power (v3.1.9.6), as we have described elsewhere (29). The analysis implied that n = 5 animals per group would provide 95% power (*p* < 0.05) to detect a 30% difference between groups; therefore, n = 5 mice or n = 4 islets/cell preparation for the *in vitro* experiments per group to avoid unpredictable experimental events.

### Statistics

The results are means ± SD for the indicated number of observations. The random significance of differences in normally distributed data sets (Shapiro-Wilk test) was analyzed by Student´s *t* test or where applicable, analysis of variance followed by Tukey-Kramers’ multiple comparisons test. *p* value < 0.05 was considered significant.

## Data availability

The RNA-seq dataset is available at https://www.pnas.org/doi/10.1073/ pnas.1402665111, where the accession number for the deposited data is GEO: GSE50398. The remaining data are available within the article and its Supplementary information.

## Supporting information

This article contains supporting information.

## Conflict of interests

Albert Salehi, PhD reports was provided by Lunds universitet. If there are other authors, they declare that they have no known competing financial interests or personal relationships that could have appeared to influence the work reported in this paper.
