# Supplementary material for: Increased ADORA1 expression in pancreatic islets coincides with insulin secretory defects in human type 2 diabetes
Source: J Biol Chem. 2026 Aug 27;302(10):113501. doi: 10.1016/j.jbc.2026.113501 (PMC13628652; doi:10.1016/j.jbc.2026.113501)
Supplement: Supplementary file 1 [file mmc1.pdf]

Supplementary Materials including Figures and tables

Increased ADORA1 expression in pancreatic islets coincides with insulin secretory defects in human type 2 diabetes

Israa Mohammad Al-Amily[1*], Fariborz Parandeh[1*], Stefan Amisten[1], Pontus Dunér[2], Sandra Meidute-Abaravisiene[1,3], Eva Grapengiesser[4], Bo Hellman[4,5] and Albert Salehi[1]

Measurements of cytoplasmic Ca^{2+} oscillations in isolated β-cells

Islets were isolated with the aid of collagenase from the splenic part of the pancreas from *ob/ob* mice sacrificed by decapitation. These islets contain N90% β-cells, which have a normal secretory response to glucose (61). Single cells and small aggregates were prepared by shaking the islets in a Ca^{2+}-deficient medium and allowed to attach to circular coverslips during 2-5 days culture at 37 °C in RPMI 1640 medium supplemented with 10% fetal calf serum, 100 IU/ml penicillin, 100 µg/ml streptomycin and 30 µg/ml gentamicin. The identification of the β-cells was based on their large size and low nucleo-cytoplasmic volume ratio compared with the islet cells secreting glucagon and somatostatin (61, 62). After culture the cells were loaded during 30-40 min with 0.5 µM fura-2 acetoxymethyl ester in the presence of 3 mM glucose. The coverslips with the attached cells were then washed and used as exchangeable bottoms of an open chamber (9 mm i.d.) connected to a two-channel peristaltic pump. The cells were superfused (0.8 ml/min) in the presence of 20 mM glucose, 20 nM glucagon and 50 µM methoxyverapamil with a medium containing 0.5 mg/ml bovine serum albumin and (in mM) 125 NaCl, 4 KCl, 1.2 $MgCl_2$, 2.6 $CaCl_2$ and 25 HEPES, with pH adjusted to 7.40 with NaOH. The studies were performed with an inverted microscope (Nikon Diaphot) placed in a climate box maintained at 37 °C. The microscope was equipped for epifluorescence fluorometry with a 400-nm dichroic mirror and a 40× Fluor oil immersion objective. A 75 W xenon arc lamp, combined with 340 and 380 nm interference filters (10-13 nm half-bandwidth), was used for excitation. Images were collected through a 30-nm half-bandwidth filter at 510 nm with an intensified CCD camera (Extended ISIS-M, Photonic Science, Robertsbridge, UK). Pairs of 340 and 380 nm images, consisting of 10–16 accumulated video frames, were captured and followed by a delay resulting in measuring cycles of 6.8 s. The specimens were illuminated only during image capture, and excitation light was minimized by

means of neutral density filters. Ratio frames were calculated after background subtraction, and the cytoplasmic Ca^{2+} concentration was estimated as previously described (31).

Measurements of hormone release from the perfused pancreas

For the situ perfusion of pancreas, the animals were anesthetized with midazolam (0.4 mg/25 g body weight) and fentanyl (0.02 mg/25 g body weight) and kept on a heating pad during the entire experiment. The abdominal cavity was opened, and arteries (renal, hepatic and splenic) were ligated, and the aorta was tied off above the level of the pancreatic artery. Pancreas was perfused at a rate of 1 ml/min via a silicone catheter placed in aorta with Krebs-Ringer bicarbonate buffer supplemented with 2% BSA and 10 mM HEPES. After 10 min of infusion the glucose concentration was raised from 3.3 to 16.7 mM. Effluent from the portal vein was collected in heparinized vials, followed by centrifugation and storage of the medium at -20 °C. Samples were taken for analysis at 60s intervals except for the first five minutes after raising glucose when the interval was 30s. Medium concentrations of insulin [32] and glucagon [33, 34] were measured with radioimmunoassay.

Insulin secretion from isolated human islets

Isolated human pancreatic islets from non-diabetic (ND) and T2D subjects were provided by the Nordic Network for Clinical Islet Transplantation (Olle Korsgren, Uppsala University, Sweden). Prior to the our experiments the islets had been cultured at 37°C (95% O2 and 5% CO2) between 1-5 days in CMRL 1066 (ICN Biomedicals, Costa Mesa, CA, USA) supplemented with 10 mM HEPES, 2 mM l-glutamine, 50 µg/ml gentamicin, 0.25 µg/ml fungizone (Gibco, BRL, Gaithersburg, MD,USA), 20 µg/ml ciprofloxacin (Bayer Healthcare, Leverkusen, Germany),10 mM nicotinamide and 5.6 mM glucose. The islets had 70–90% purity when they arrived and they were then hand-picked under a stereomicroscope prior to use. All procedures were approved by the ethical committees at Uppsala and Lund University.

After washing islets in Hank`s buffer they were transfer to a vial (12 per each vial) containing an ordinary Krebs-Ringer bicarbonate (KRB) buffer supplemented with 10 mM HEPES and 1 mM glucose. The islets were gassed with 95% O2 and 5% CO2 to obtain constant pH and oxygenation and preincubated at for 30 min. Thereafter, the preincubation media were removed and the islets were incubated for 60 min at 1 or 16.7 mM glucose ± indicated agents. After incubation an aliquot of the medium was removed for the assay of insulin.

Supplementary Figures

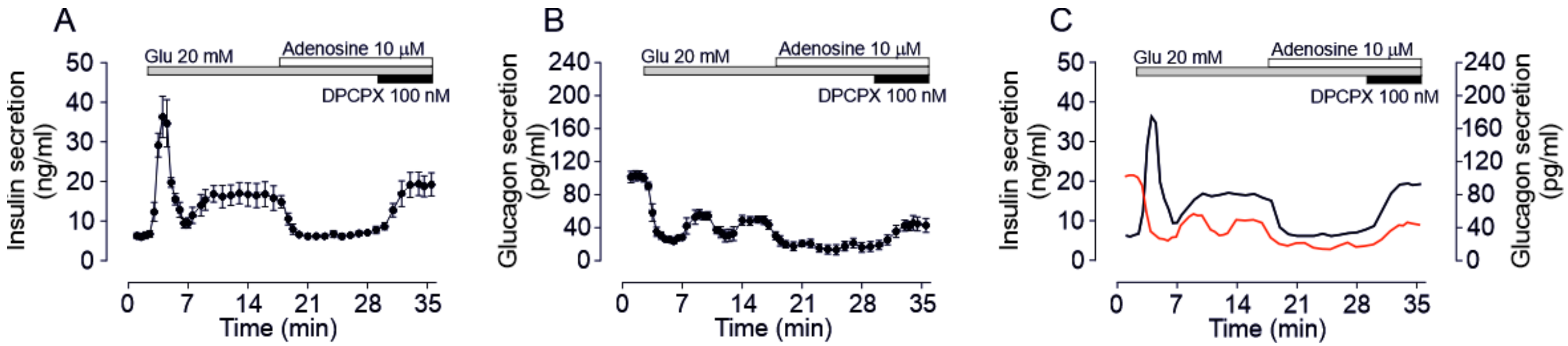

Figure Supplementary 1. Effects of Adora1 blocker (DPCPX) on the adenosine-induced suppression of insulin and glucagon secretion from perifused C57BL/6 mouse islets. Batches of 50 islets were perifused with KRB buffers containing 1 mM glucose followed by raising glucose to 20 mM with addition of adenosine (10 µM) and DPCPX (100 nM) as indicated. The insulin (A) and glucagon (B) were measured in 30-sec fractions of the perifusate. The suppressive effect of adenosine on hormone secretion was attenuated by DPCPX. The temporal relationship between insulin (black line) and glucagon secretion (red line) is illustrated in (C). Mean ± SD for three experiments performed at different occasions are shown.

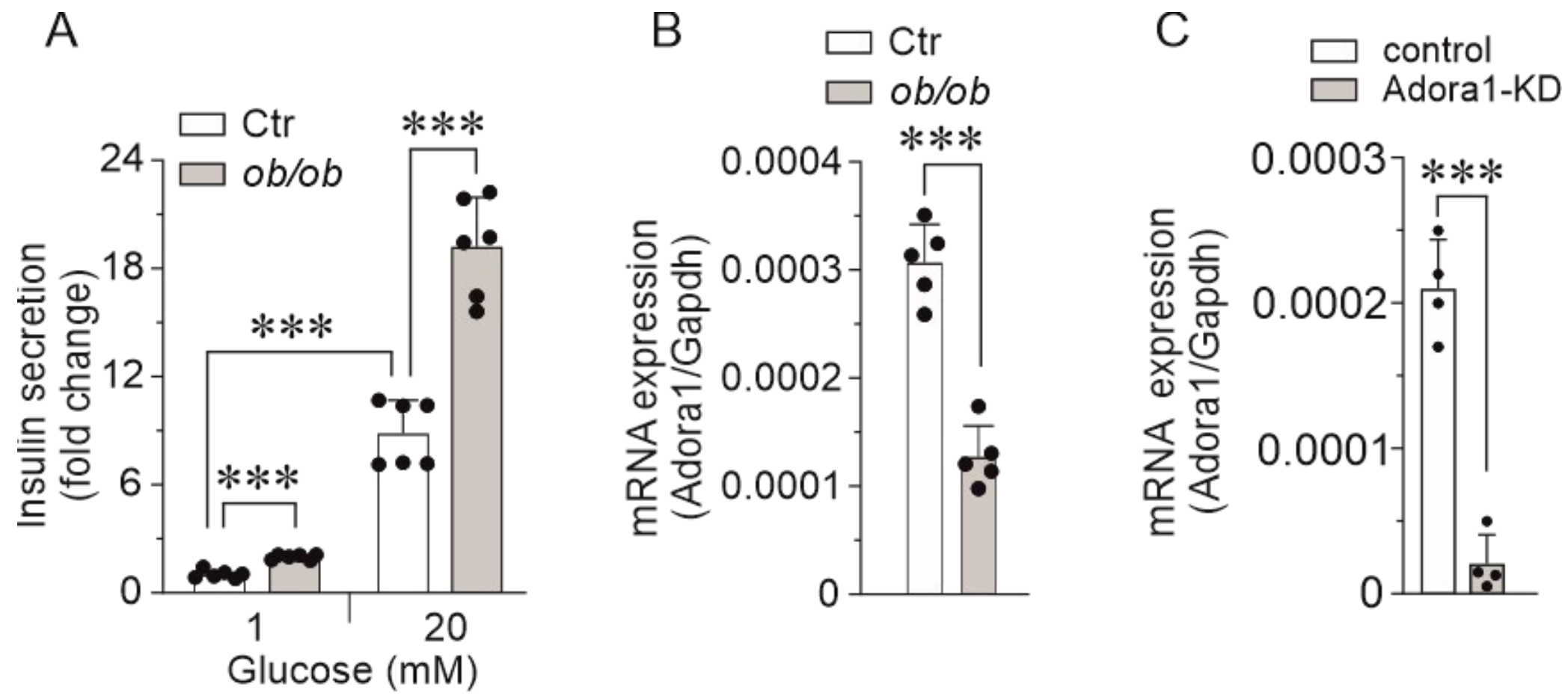

Figure Supplementary 2. Hypersecretion of insulin by *ob/ob* islets coincides with lower expression of Adora1. (A) Glucose-stimulated Insulin secretion from islets taken from C57BL/6 control and *ob/ob* (obese) mice and incubated at 1 mM or 16,7 mM glucose. Results are presented as means±SD for 6 different animals in each group. *** P<0.001. (B) qPCR analysis of Adira1 mRNA expression in control and *ob/ob* mice. Mean±SD for n=5 mice in each group are shown. (C) qPCR analysis of Adora1 mRNA expression in control and Adora1-KD mice. The results are mean±SD for n=4 mice in each group. *** P<0.001 denotes unpaired Student´s t-test analysis for each glucose concentration in control and *ob/ob* (A and B) as well as unpaired Student´s t-test between control and Adora1-KD (C).

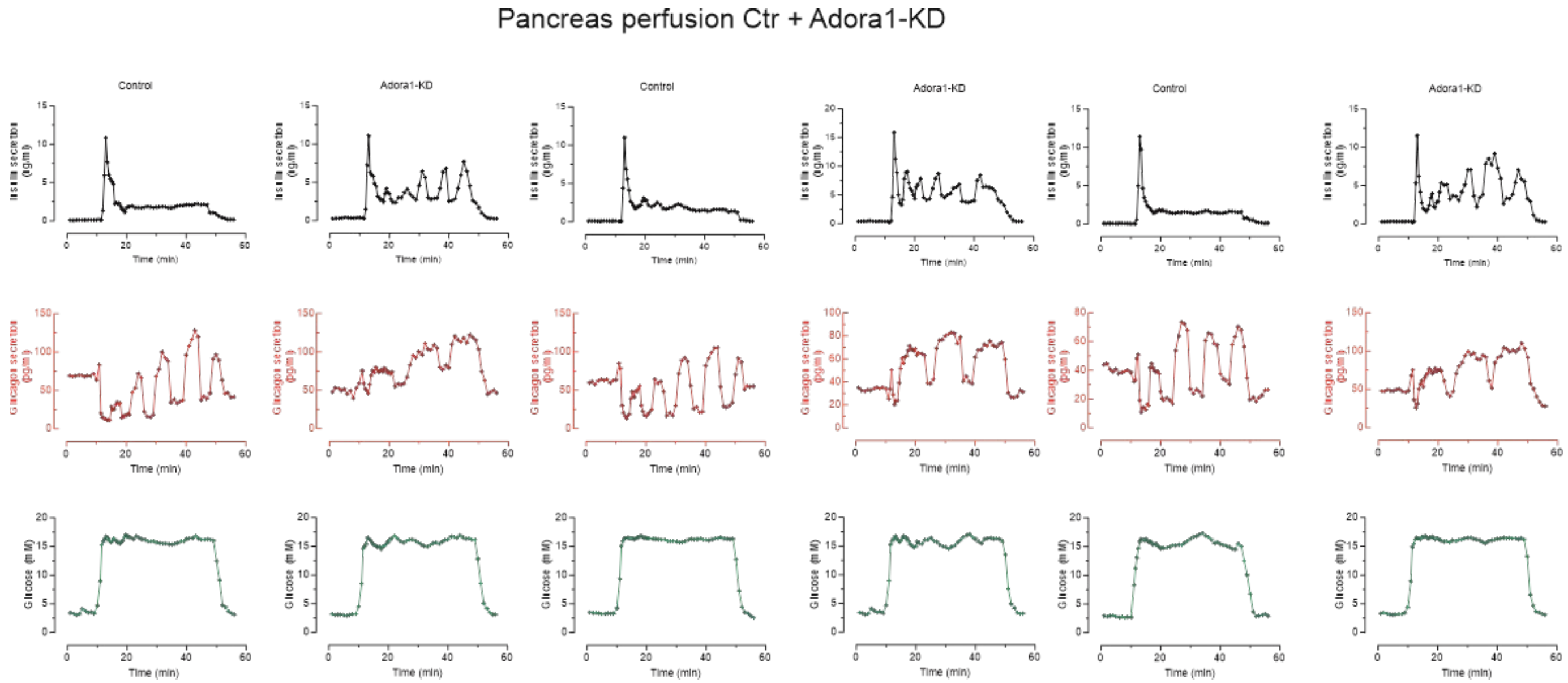

Figure Supplementary 3. Dynamics of insulin and glucagon secretion from perfused pancreas in individual control (+/+) and individual Adora1 (-/-) mice. (A) Insulin and (B) glucagon secretion measured in control and Adora1 (-/-) by pancreas-perfusion before and after rise in glucose concentration from 3.3 to 16.7 mM. (C) The glucose concentrations in each perfusate sample analyzed by glucose oxidase method. The analyzed data are from 4 mice in each group as shown in our previous publication i.e. reference (11).

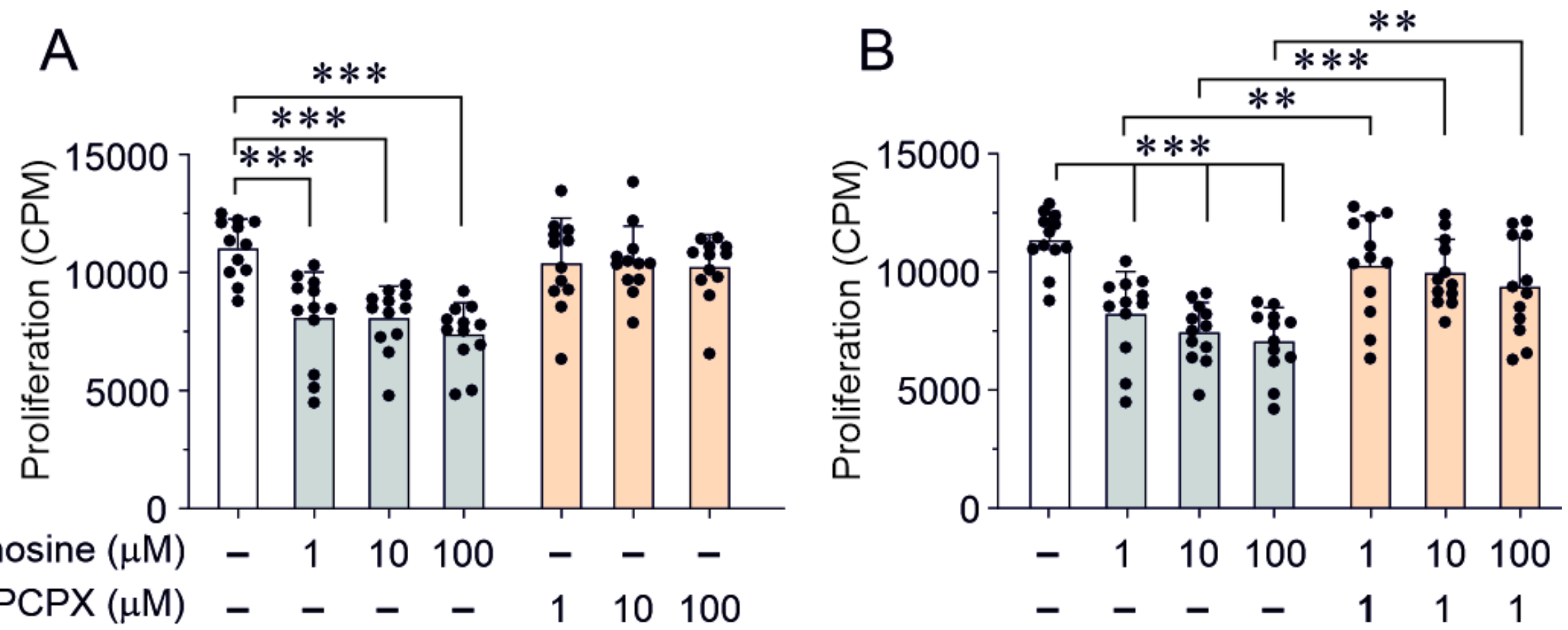

Figure Supplementary 4. Effect of adenosine or DPCPX on cell proliferation. The INS-1 832/13 Cells were cultured at 5 mM glucose with different concentrations of adenosine or DPCPX or different concentration of adenosine + DPCPX 1 μM. Means ± SD for n=12 observations are shown. **P<0.01, ***P<0.001 (Significance was assessed by unpaired Student´s t-test between control and each treated group).

Supplementary table 1. Primers used for quantitative real-time PCR of human and mouse adenosine receptors.

Gene symbol	Human primer assay (Qiagen)	Mouse primer assay (Qiagen)
ADORA1	QT01531635	QT00301119
ADORA2A	QT00210245	QT02527308
ADORA2B	QT00029911	QT00257558
ADORA3	QT00041181	QT01068347
GAPDH	QT01192646	QT01658692

Supplementary table 2. mRNA expression levels of genes strongly associated (Pearson Correlation coefficient > 0.70) with the mRNA expression level of ADORA1 as identified by RNA-seq of 89 human islet preparations. The Ingenuity Pathways Analysis (IPA) software was used to assign to each gene functions relevant for β-cell biology.

Gene 1	Gene 2(symbol)	Correlation(Pearson's r)	Percentage rank of Gene 2 (*low percentage means high expression*)	Gene 2, Average log2 CPM
ADORA1	ERBB2	0,84181869	20,28549604	5,578556267
ADORA1	KCNK5	0,825615306	32,5026258	4,60619479
ADORA1	RHOV	0,82219344	50,79728827	2,831349938
ADORA1	PPAP2C	0,816218985	37,17654922	4,228514812
ADORA1	PMEPA1	0,803140955	2,654444763	7,852665372
ADORA1	EPHB6	0,802606766	53,72863554	2,443185754
ADORA1	CLDN10	0,80015224	29,70018142	4,820190313
ADORA1	TRPV6	0,796059605	27,47063879	4,99116184
ADORA1	RBPMS	0,789007684	26,2961902	5,082366225
ADORA1	LYPD1	0,786876212	54,63095579	2,317804145
ADORA1	SYT12	0,785231108	65,23918648	0,744473505
ADORA1	ABCC3	0,784882804	8,722429103	6,670727816
ADORA1	IFFO2	0,784570317	27,25580063	5,01209572
ADORA1	KIAA1522	0,783123201	7,337916547	6,865696149
ADORA1	IGSF3	0,780420325	32,30688437	4,623129283
ADORA1	TSPAN15	0,779888949	25,26974124	5,164823312
ADORA1	LRG1	0,774800266	37,92132149	4,16270492
ADORA1	SPINT1	0,772861277	4,258569655	7,422330474
ADORA1	SLC52A3	0,77082441	67,71221236	0,405274962
ADORA1	FBLIM1	0,769860266	22,39568414	5,401407639
ADORA1	RGS3	0,769785225	31,20882269	4,699071507
ADORA1	GGT6	0,767356035	51,6518667	2,719521593
ADORA1	MYRF	0,765072814	11,09519717	6,396045376
ADORA1	PPP1R1B	0,762977004	54,34450492	2,35113012
ADORA1	EPHA1	0,762163286	50,19096725	2,9200274

ADORA1	EVC	0,76200968	46,20452592	3,370569941
ADORA1	ASAP3	0,761540345	44,51446577	3,536150455
ADORA1	SHMT1	0,761441989	51,7807696	2,703752397
ADORA1	SMO	0,761341426	51,20786785	2,777477935
ADORA1	ELF3	0,761263198	5,065406283	7,261934876
ADORA1	CNN2	0,758846233	38,29848181	4,130424632
ADORA1	EPHB4	0,758271985	35,64403705	4,350324126
ADORA1	EFNA4	0,755176298	58,85610618	1,694684758
ADORA1	KIF1C	0,75129674	6,163467965	7,027758552
ADORA1	IDH2	0,745871712	18,71479041	5,702315807
ADORA1	CDH3	0,745415683	45,1160126	3,480855169
ADORA1	CAMKK2	0,745124311	31,58120882	4,674690728
ADORA1	NEK6	0,744851958	30,36856679	4,768981115
ADORA1	CTSH	0,743622752	19,29246634	5,651861192
ADORA1	CGN	0,743590373	15,53041153	5,971709892
ADORA1	GGT1	0,743559407	43,71240332	3,614062068
ADORA1	HNF1B	0,741835703	37,91654731	4,162843571
ADORA1	CSMD2	0,740674926	56,91301442	1,992411671
ADORA1	SFRP5	0,739381685	50,39625704	2,889031104
ADORA1	FGD3	0,738474604	61,08087463	1,358974521
ADORA1	KCNQ1	0,735067242	46,50529934	3,34458042
ADORA1	ADCY5	0,733351767	36,34584169	4,292140876
ADORA1	ESRP2	0,73260341	34,72739425	4,4330492
ADORA1	LGALS9	0,732415471	43,00105032	3,68340481
ADORA1	TRPV4	0,73169031	65,3871861	0,72023895
ADORA1	IL27RA	0,729986136	55,99637162	2,126755959
ADORA1	IL4R	0,729785194	16,59027977	5,880990859
ADORA1	SLC8B1	0,727748675	41,87911773	3,799277914
ADORA1	EPN3	0,727424494	59,52926573	1,600694564
ADORA1	SDC3	0,727350633	39,38222095	4,025302764
ADORA1	PCED1B	0,727001123	68,57156498	0,289909422
ADORA1	ACAA1	0,726234234	25,98586842	5,107947528
ADORA1	GOLM1	0,72569393	3,637926096	7,555321602
ADORA1	FUT6	0,725578758	63,96925427	0,94182901
ADORA1	ST6GALNAC2	0,724610326	56,34011267	2,073692422
ADORA1	CLGN	-0,723242816	19,03943474	5,673614714

ADORA1	RIPK3	0,718463173	56,73159553	2,012805363
ADORA1	PDZK1IP1	0,714588595	36,58455075	4,278818076
ADORA1	ONECUT1	0,714513461	53,42308794	2,486440287
ADORA1	ACSS1	0,713935048	43,20156593	3,662129189
ADORA1	TSPAN14	0,713867309	14,4705433	6,063781822
ADORA1	TMEM102	0,71208364	54,56411725	2,327091379
ADORA1	RAB39B	-0,711417649	27,69025112	4,978060062
ADORA1	TMEM63A	0,711191437	26,7545116	5,047723264
ADORA1	PROM2	0,710358029	46,11859066	3,380059697
ADORA1	C3orf14	-0,710314709	24,7445813	5,204412666
ADORA1	PALM3	0,710171138	54,12489258	2,378117112
ADORA1	LAD1	0,710076105	14,00267354	6,104476461
ADORA1	KRT8	0,709247408	0,386708679	9,665772123
ADORA1	CDC42EP3	-0,709147358	9,562685	6,579212946
ADORA1	ZNF569	-0,708074071	45,34994748	3,452395605
ADORA1	PTPRF	0,707437639	2,10063974	8,074693265
ADORA1	FGFR2	0,707337056	45,07781915	3,483324595
ADORA1	BMI1	-0,706948956	11,2145517	6,384825868
ADORA1	TBC1D16	0,706719599	16,41363506	5,899336659
ADORA1	SOX9	0,706300727	26,18638404	5,090537081
ADORA1	SCNN1A	0,706164357	20,57672109	5,55461947
ADORA1	LITAF	0,705889321	8,817912728	6,660906257
ADORA1	CAPN6	0,705782872	51,08373914	2,795260362
ADORA1	C1RL	0,704145977	28,27747541	4,935207861
ADORA1	GRB7	0,702519603	43,55485534	3,627177843
ADORA1	GPR157	0,700547814	49,81380693	2,962768861
